# RADA16 and SAAP148 Peptide‐Modified Collagen Self‐Assembled Hydrogels for Accelerated Healing of Infected Wounds

**DOI:** 10.1002/advs.202519504

**Published:** 2025-11-16

**Authors:** Qian Liu, Jiawei Wu, Ho‐Pan Bei, Yufei Chen, Yifan Zhang, Xueliang Peng, Fulin Chen, Xin Zhao, Zhuoyue Chen

**Affiliations:** ^1^ Key Laboratory of Resource Biology and Biotechnology in Western China Ministry of Education Provincial Key Laboratory of Biotechnology College of Life Sciences Northwest University 229 North Taibai Road Xi'an Shaanxi Province 710069 China; ^2^ Department of Applied Biology and Chemical Technology The Hong Kong Polytechnic University 11 Yuk Choi Rd Hung Hom Hong Kong SAR 999077 China; ^3^ Research Institute for Intelligent Wearable Systems The Hong Kong Polytechnic University Hung Hom Kowloon Hong Kong SAR 999077 China

**Keywords:** anti‐bacterial peptides, infected wound healing, multiscale fibrous hydrogel, self‐assembly peptides

## Abstract

Infected wounds suffer from limited self‐healing, persistent bacterial infections, prolonged inflammation, and oxidative wound microenvironment. While anti‐bacterial peptides such as SAAP148 demonstrate remarkable efficacy against drug‐resistant pathogens, their clinical application is hindered by rapid inactivation and uncontrolled burst release. To address these limitations, collagen type I (Col I) is integrated with self‐assembling peptide RADA16 to develop a novel self‐assembled nano‐micro structured hydrogel (Col I‐RADA16, CR) without chemical cross‐linkers. This unique design leverages the micron‐scale porous structure of Col I and the nanofibrous architecture of RADA16, resulting in a hydrogel with excellent mechanical properties, sustained SAAP148 release, and enhanced bioactivity. CR not only promotes fibroblast adhesion, migration, and proliferation, but when loaded with SAAP148 (Col I‐RADA16‐SAAP148, CRS), effectively inhibits bacterial infection, enhances macrophage polarization and accelerates wound healing in vivo. Importantly, histological and immunohistochemical analyses revealed that the CRS hydrogel significantly enhances regeneration of skin appendages (e.g., hair follicles and glands) by action of CK5 and CK14 in the ERBB/MAPK, mTOR/PI3K‐Akt, JNK/p38 MAPK signaling axes, significantly surpassing the performance of traditional collagen or gelatin sponges. This innovative dual‐scale design and cross‐linker‐free fabrication strategy offers a versatile and clinically translatable platform for infected wound healing, addressing critical limitations in current wound care technologies.

## Graphical abstract

Collagen type I (Col I) is integrated with self‐assembling peptide RADA16 and loaded with anti‐bacterial peptide SAAP148 to develop a self‐assembled nano‐microstructure hydrogel (Col I‐RADA16‐SAAP148, CRS) without chemical cross‐linkers. CRS exhibits excellent mechanical properties, sustained SAAP148 release to inhibit bacterial infection, macrophage polarization and enhanced skin appendage regeneration by action of CK5/14, surpassing performance of traditional collagen or gelatin sponges.

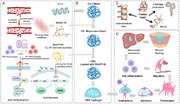

## 1 Introduction

Skin is the body's largest organ, serving as a critical barrier against external threats to prevent infections, fluid loss, and electrolyte imbalance.^[^ ^1^ ^]^ However, when compromised by physical, chemical, or microbial insults, the skin's protective function is disrupted, leading to open wound, microbial colonization, and subsequent complications such as pain, swelling, and redness.^[^ ^2^ ^]^ Severe wounds often result in bleeding and infection, which can escalate to life‐threatening conditions if not properly managed. Despite significant advancements in wound care, the development of multifunctional biomaterials that simultaneously address hemostasis, antimicrobial activity, and skin regeneration remain a major challenge.

Traditional wound repair strategies primarily rely on skin transplantation or synthetic skin substitutes. Autologous skin transplantation, while effective, is limited by donor scarcity and secondary trauma.^[^ ^3^ ^]^ Allogeneic and xenogeneic transplants, on the other hand, pose risks of immune rejection and viral transmission.^[^ ^4^ ^]^ Synthetic skin substitutes such as Integra, Dermagraft, and Transcyte have shown promise in wound healing by providing a temporary protective barrier and promoting cell adhesion and proliferation.^[^ ^5^ ^]^ However, the manufacturing process of these synthetic skins is complex and expensive, limiting their widespread use. Most importantly, these materials often fail to fully address critical challenges such as bleeding control, infection prevention, and functional skin regeneration. Notably, they cannot fully mimic the functions of natural skin (e.g., sweat glands, hair follicles, etc.). Recent studies have highlighted the importance of multifunctional wound dressings that integrate hemostatic, antimicrobial, and regenerative properties.^[^ ^6^, ^7^, ^8^ ^]^ For instance, advanced hydrogels incorporating antimicrobial nanomaterials have demonstrated potent antibacterial activity, but are limited by their poor in vivo stability in clinical applications.^[^ ^9^, ^10^, ^11^ ^]^ Similarly, growth factor‐loaded scaffolds have shown potential in promoting tissue regeneration but are burdened with high costs and instability.^[^ ^12^ ^]^ These limitations underscore the need for innovative materials that can simultaneously address multiple aspects of wound healing.

It is a good idea to mimic the structure and function of natural tissues through nature‐derived strategies to promote functional regeneration. Collagen I (Col I) is a major component of the skin extracellular matrix (accounting for ≈80% of adult skin) which plays a dual role as a structural scaffold and a signaling molecule in wound healing.^[^ ^13^ ^]^ Its high hydrophilicity, biodegradability, and biocompatibility make it an ideal candidate for skin substitutes. However, conventional collagen‐based materials lack the mechanical strength and multifunctionality required to simultaneously control bleeding, prevent infection, and promote tissue regeneration. To overcome some of these limitations, researchers have explored supplementing collagen‐based materials with natural clotting agents such as platelet‐rich plasma (PRP). Upon contact with injured tissue, PRP triggers the release of fibrinogen and coagulation factors V, VII, XI, and XIII from platelet α‐granules, accelerating the formation of a fibrin network that evolves into a thrombus to achieve hemostasis. However, the clinical utility of PRP is constrained by challenges in its preparation, storage, transportation, and foreign body response, particularly in scenarios requiring portability (e.g., field applications or combat settings), as well as its inability to enhance the tensile strength or elasticity of clinical dressings. Meanwhile, the development of self‐assembling peptide (SAP) enabled the exploration of self‐cross‐linking matrices with various tissue niches. RADA16 can replicate the physiological effects of fibrin by forming a nanofibrous network that promotes rapid blood clotting without triggering inflammation or immune rejection.^[^ ^14^ ^]^ The SAP RADA16 has been sold as a hemostatic agent for surgery (PuraStat) and as a reagent for life science research (PuraMatrix). PuraStat is a CE‐marked class III medical device for hemostasis during surgery, especially for bleeding from small blood vessels and oozing from capillaries of the solid organs' parenchyma and vascular anastomoses.^[^ ^15^ ^]^ However, RADA16 alone as a bulk matrix (e.g., dense hydrogels) has severe limitations. i) Limited cell infiltration: bulk RADA16 hydrogels often form nanoscale pores (5 – 20 nm), which are too small for cells (typically requiring > 50 µm pores) to infiltrate, restricting cell migration and tissue integration. ii) Lack of bioactive signals: RADA16 lacks cell‐specific signaling motifs (e.g., RGD, IKVAV), relying on adsorbed ECM proteins (e.g., Col I) to mediate cell interactions, limiting cell attachment and proliferation in low‐protein environments. iii) Slow degradation: RADA16 is protease‐resistant due to its stable β‐sheet structure. This slow degradation can hinder scaffold remodeling and replacement by native tissue.

Hence, we aimed to develop a composite hydrogel that combines the structural benefits of Col I with the hemostatic efficacy of RADA16. RADA16 can bind with Col I through complementary charge interactions and intermolecular forces such as hydrogen bonding, hydrophobic interactions, or van der Waals forces. Its nano‐micro structural interactions between peptide chains serve as a good vessel for cell adhesion, as well as loading of antibacterial molecules to prevent bacterial colonization. While traditional antibiotic‐loaded dressings are increasingly ineffective due to the rise of drug‐resistant pathogens,^[^ ^16^ ^]^ antimicrobial peptides (AMPs) such as LL‐37 offering a promising alternative by targeting bacterial membranes and modulating immune responses more effectively.^[^ ^17^ ^]^ Inspired by the natural antimicrobial activity of LL‐37 and its truncated structure that can enhance the bactericidal effect, Nibbering et al. have successfully developed a novel derivative, SAAP148, by systematically randomly replacing the peptide chain of LL‐37 (to maintain the stability of the overall structure).^[^ ^18^ ^]^ Unlike traditional AMPs, SAAP148, a 24‐amino acid peptide (LKRVWKRVFKLLKRYWRQLKKPVR) has demonstrated exceptional efficacy against multidrug‐resistant bacteria, including *methicillin‐resistant Staphylococcus aureus* (MRSA) and *Acinetobacter baumannii*.^[^ ^18^ ^]^ SAAP148 is positively charged at physiological pH, allowing it to bind to the negatively charged cell membranes of both Gram‐negative and Gram‐positive bacteria (e.g., phospholipid head groups in Gram‐negative bacteria or teichoic acids in Gram‐positive bacteria). Its amphipathic structure (with alternating hydrophobic and hydrophilic regions) enables insertion into the bacterial lipid bilayer, disrupting membrane integrity. This leads to leakage of cellular contents and ultimately bacterial death.^[^ ^19^, ^20^ ^]^ Simultaneously, the positively charged SAAP148 can directly neutralize LPS (a negatively charged bacterial toxin) and form stable complexes. This binding blocks LPS–induced activation of NF–κB and MAPK signaling pathways and reduces the release of pro–inflammatory cytokines (TNF–α, IL–6, IL–1β).^[^ ^21^, ^22^ ^]^ The simple structure of SAAP148 in absence of disulfide bonds enhances its stability and simplifies synthesis. With significantly lower hemolytic activity and cytotoxicity compared to other antimicrobial peptides, SAAP148 emerges as a highly promising agent against a broad spectrum of pathogens. However, the clinical application of SAAP148 is hindered by its rapid inactivation and uncontrolled release in vivo, which could be expectedly remedied through peptide‐cross‐linking and entanglement with nano‐micro structured hydrogels.

In this study, we designed a novel self‐assembled nano‐micro structured hydrogel (CRS) by combining collagen I (Col I) with RADA16 and SAAP148. This innovative design leverages the micro‐scale porous structure of Col I and the nanofibrous architecture of RADA16, resulting in a hydrogel with excellent mechanical properties, sustained SAAP148 release, and enhanced bioactivity. The CRS hydrogel not only promotes fibroblast adhesion, migration, and proliferation, but also effectively inhibits bacterial infection and accelerates wound healing in vivo. Importantly, histological and immunofluorescence analyses revealed that CRS hydrogels significantly enhance the regeneration of skin appendages (e.g., hair follicles and glands) possibly through CRS‐induced expression of CK14. CK14 can promote skin regeneration by facilitating the secretion of epidermal growth factor (EGF)‐like domain molecules and genes that participate in cell‐cell junction regulation, mitosis, and transcription factors (TFs).^[^ ^23^ ^]^ This innovative dual‐scale (nano‐micro) design and cross‐linker‐free fabrication strategy represent a significant breakthrough in wound care technology. By addressing the limitations of existing materials such as poor mechanical properties, uncontrolled drug release, and lack of multifunctionality, our hydrogel offers a versatile and clinically translatable platform for infected wound healing. The integration of Col‐I, RADA16, and SAAP148 not only provides a comprehensive solution for hemostasis, antimicrobial activity, and tissue regeneration but also opens new avenues for the development of next‐generation self‐assembling injectable biomaterials for repair of intervertebral disc, nerve, and articular cartilage damage.

## 2 Results

### 2.1 Structure of the Col I, CR, and CRS

**Figure 1:**
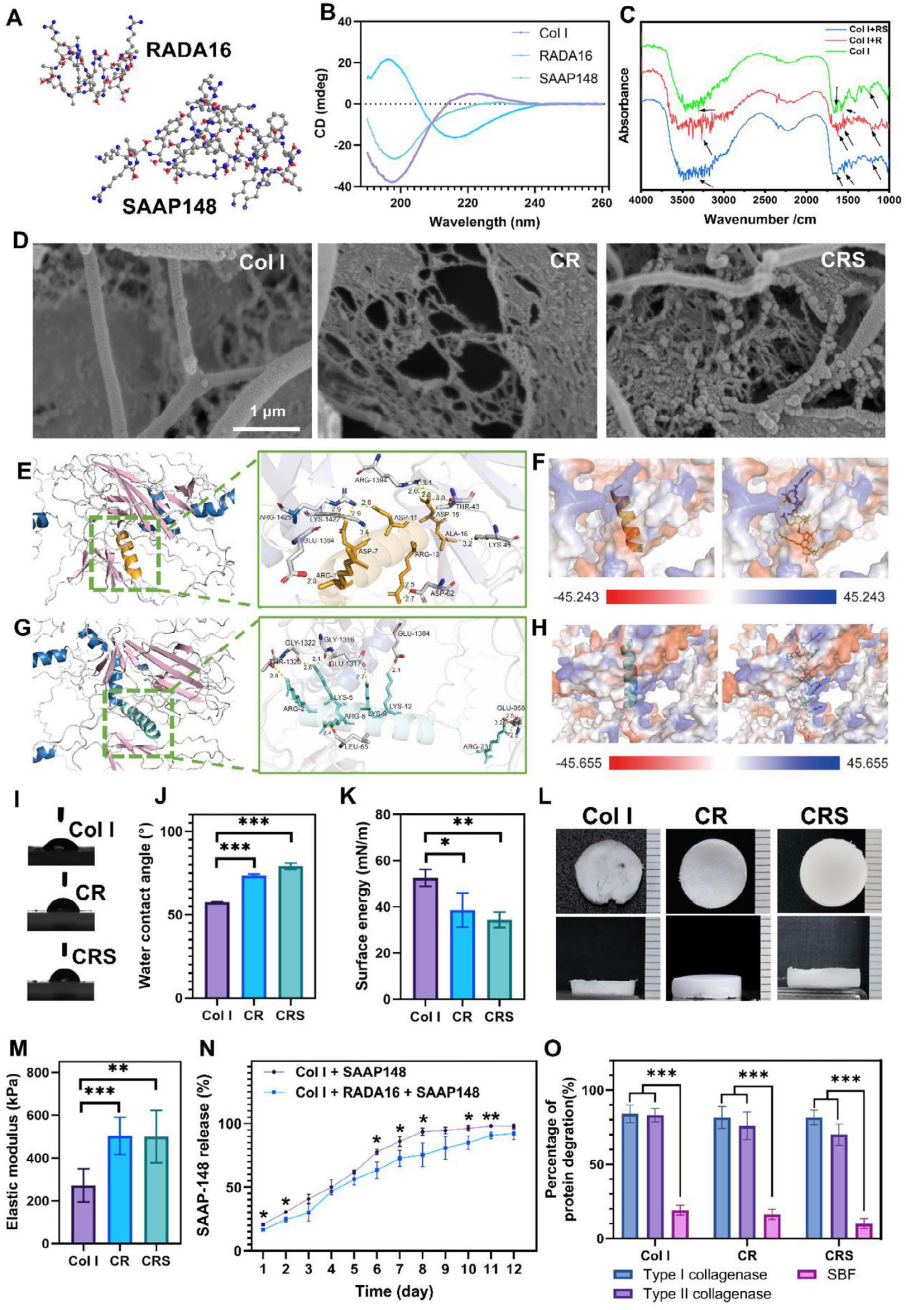
Morphology and physicochemical properties of the Col I, CR, and CRS. A) Chemical structures of RADA16 and SAAP148 peptides; B) Circular dichroism of the Col I, RADA16 and SAAP148; C) FTIR spectra; D) cryo‐electron microscopy; E) Molecular docking of Col I and RADA16; F) Electrostatic potential of the Col I bind with RADA16. Negative electrostatic potential is presented in red while positive electrostatic potential is presented in blue; G) Molecular docking of Col I and SAAP148; H) Electrostatic potential of the Col I binding with SAAP148. Negative electrostatic potential is presented in red while positive electrostatic potential is presented in blue; I,J) Water contact angle and K) Surface free energy evaluation of Col I, CR, and CRS hydrogels; L) The general morphology of the lyophilized Col I, CR, and CRS scaffolds; M) Elastic modulus of Col I, CR, and CRS scaffolds; N) The comparison of the SAAP148 release rate from Col I sponge and CRS scaffold; O) Degradation rates of Col I sponge, CR and CRS scaffold under different collagenase environments. Mean ± SD, *p < 0.05, **p < 0.01, ***p < 0.001, *n* = 3.

The mass spectrometry (MS) and high‐performance liquid chromatography (HPLC) identification of RADA16 (Figure S1, Supporting Information) and SAAP148 (Figure S2, Supporting Information) indicated that the molecular weight and purity met the experimental requirements. We simulated the tertiary structures of the two short peptides, RADA16 and SAAP148, based on their amino acid sequences (**Figure** **1**A). The CD results indicated that Col I exhibited a large negative peak in the 190–210 nm range and a small positive peak at 220 nm, suggesting the presence of a distinct triple‐helical structure. RADA16 showed a large negative peak in the 200–220 nm range, indicating a typical β‐sheet structure. The SAAP148 peptide, with its simpler structure, displayed only a negative peak in the 190–210 nm range (Figure 1B). The analysis of the FTIR spectra indicated that Col I, CR, and CRS were all capable of identifying the characteristic absorption peaks associated with collagen. These included the protein amide A peak at 3288 cm^−1^, the amide I peak at 1641 cm^−1^, the amide II peak at 1543 cm^−1^, and the amide III peak at 1236 cm^−1^ (Figure 1C). These data suggested that Col I retained the triple helix structure of collagens. Self‐assembled structures of the Col I, CR, and CRS were observed by a cryo‐SEM (Figure 1D). Both Col I and CR can self‐assemble to form fibrous structures. It was noteworthy that micro‐scale fibers were found in Col I and a large number of nano‐scale fibers were found in CR (Figure 1D). Some nanoparticles were observed in cryo‐SEM after the addition of SAAP148 (CRS). Since SAAP148 did not conventionally self‐assemble into fibers, we speculated that the nanoparticles on the fibers were SAAP148.

To further elucidate the binding interactions between Col I and RADA16 and SAAP148, molecular docking studies were performed using Alphafold3. The results showed that RADA16 fits well with Col I and forms multiple non‐covalent interactions (Figure 1E). Specifically, RADA16 formed multiple hydrogen bonds with residues such as Arg1425, Lys1422, Thr43, and Ala16. SAAP148 also fit well with Col I and formed multiple non‐covalent interactions (Figure 1G). SAAP148 formed multiple hydrogen bonds with residues such as Lys5, Arg6, Leu65, and Glu958. Furthermore, as shown in Figures 1F,H, electrostatic potential maps indicated that both RADA16 and SAAP148 bound well to the region between the negatively and positively charged regions of Col I.

### 2.2 Properties of the Col I, CR, and CRS Scaffolds

To evaluate the cytocompatibility of the materials, the water contact angle and surface energy (Figure 1I–K) of the Col I, CR, and CRS films were tested. The results showed that the water contact angle of CR and CRS significantly increased (***p < 0.001), while the surface energy of CR (*p < 0.05) and CRS (**p < 0.01) significantly decreased compared with Col I film (Figure 1I,J). It may be due to the change of functional groups on the surface of Col I (Figure 1C), which have different affinities for water molecules resulting in a decrease in the hydrophilicity of Col I. Nevertheless, even if the introduction of the RADA16 peptide results in a decrease in the water contact angle, the CR and CRS remain hydrophilic and favorable for cell adhesion. After being lyophilized, the Col I, CR, and CRS all exhibited loose and spongy characteristics (Figure 1L). SEM images (Figure S3, Supporting Information) of the Col I sponge showed high porosity and large pore size (≈ 50 µm). After mixing with RADA16 and SAAP148 peptide, the CR and CRS sponge not only had porous structure but also formed nanofibers (Figure S3, Supporting Information). The dual‐scale architecture combining collagen networks (≈ 50 µm pore size) with RADA16 nanofibers (8 – 12 nm diameter) significantly enhanced mechanical performance, as evidenced by a 185% increase in elastic modulus for cross‐linked composites (CR: 503.66 ± 87.78 kPa, ***p < 0.001; CRS: 500.22 ± 123.29 kPa, **p < 0.01) relative to pure collagen I (271.63 ± 77.22 kPa, Figure 1M). This enhancement arises from hierarchical fiber entanglement where collagen microfibrils (50 – 200 nm) mechanically interlock with β‐sheet‐rich RADA nanofilaments (Figure 1D), creating synergistic energy dissipation pathways through: i) Fiber bridging: Nanofibers spanning collagen micropores; ii) Shear‐lag transfer: Stress redistribution at micro‐nano interfaces; iii) Covalent reinforcement: Tyrosine‐mediated cross‐linking between collagen triple helices and RADA domains. The elastic modulus of both CR and CRS nanocomposites increased by 1.85 times compared to collagen controls, which demonstrated the superior load‐bearing capacity of nanocomposites under stress (Figure 1M).

### 2.3 Release Rate of the SAAP148 from the Nano‐Micro Hydrogels

The kinetic release behavior of the Col I hydrogel and Col I& RADA hydrogel over 12 days is shown in Figure 1N. On days 1, 2, 6, 7, 8, 10, and 11, the percentage of the released SAAP148 from CR hydrogel was significantly lower than that of the Col I hydrogel (*p < 0.05, Figure 1N), which indicated that the CR hydrogel was more suitable as a sustained‐release system of the SAAP148 compared to the Col I hydrogel. We analyzed that the slower release of SAAP148 observed in CRS from the fact that RADA16 formed an interconnected nano‐network with Col I (Figure 1D) to form a denser CRS matrix, which enhanced the physical retention through structural constraints, while electrostatic interactions between SAAP148 and the alternating cationic/anionic residues in the RADA peptide generated additional binding affinity. The combined effect of these structural and electrostatic factors effectively attenuated the release of SAAP148.

### 2.4 Stabilities of the Col I, CR, and CRS Hydrogels

During the molecular dynamics simulations, the RMSD values were used to assess the deviation of protein molecules from their reference positions, thereby reflecting the stability of the simulation system. Analysis of RMSD trends over time revealed that antimicrobial peptides exhibited greater stability compared to Col I, which showed significant fluctuations with a continuous increase in its RMSD value over the course of the simulation (Figure S4A, Supporting Information). Furthermore, an analysis of the Rg values throughout the simulation period indicated that Col I maintained a relatively small Rg, suggesting a more compact structure. In contrast, the antimicrobial peptides had larger Rg values, indicating a more extended conformation. However, both Col I and the antimicrobial peptides displayed substantial fluctuations in their Rg values, pointing to multiple conformational changes within their structures (Figure S4B, Supporting Information).

The degradation behavior of the composite hydrogels was further examined using collagenase and SBF to assess their biostability in vivo. Quantitative analysis demonstrated that collagenase type I induced significant degradation of Col I (84.081 ± 5.892%, *n* ═ 3), CR (81.573 ± 7.408%, *n* ═ 3), and CRS (81.612 ± 5.050%, n═3, Figure 1O). Compared to SBF controls, collagenase type II treatment also resulted in markedly higher degradation rates for Col I (83.252 ± 4.457%, *n* ═ 3), CR (75.924 ± 9.209%, *n* ═ 3), and CRS (70.068 ± 7.227%, *n* ═ 3) (***p < 0.001, Figure 1O). Collagenase accelerated the degradation of the composite hydrogels containing collagen, and it can be hypothesized that Col I, CR and CRS were mainly degraded by collagenase in vivo and were not limited to one collagenase.

### 2.5 Effect on Cell Viability

#### 2.5.1 Fibroblast Adhesion on the Col I, CR, and CRS

**Figure 2:**
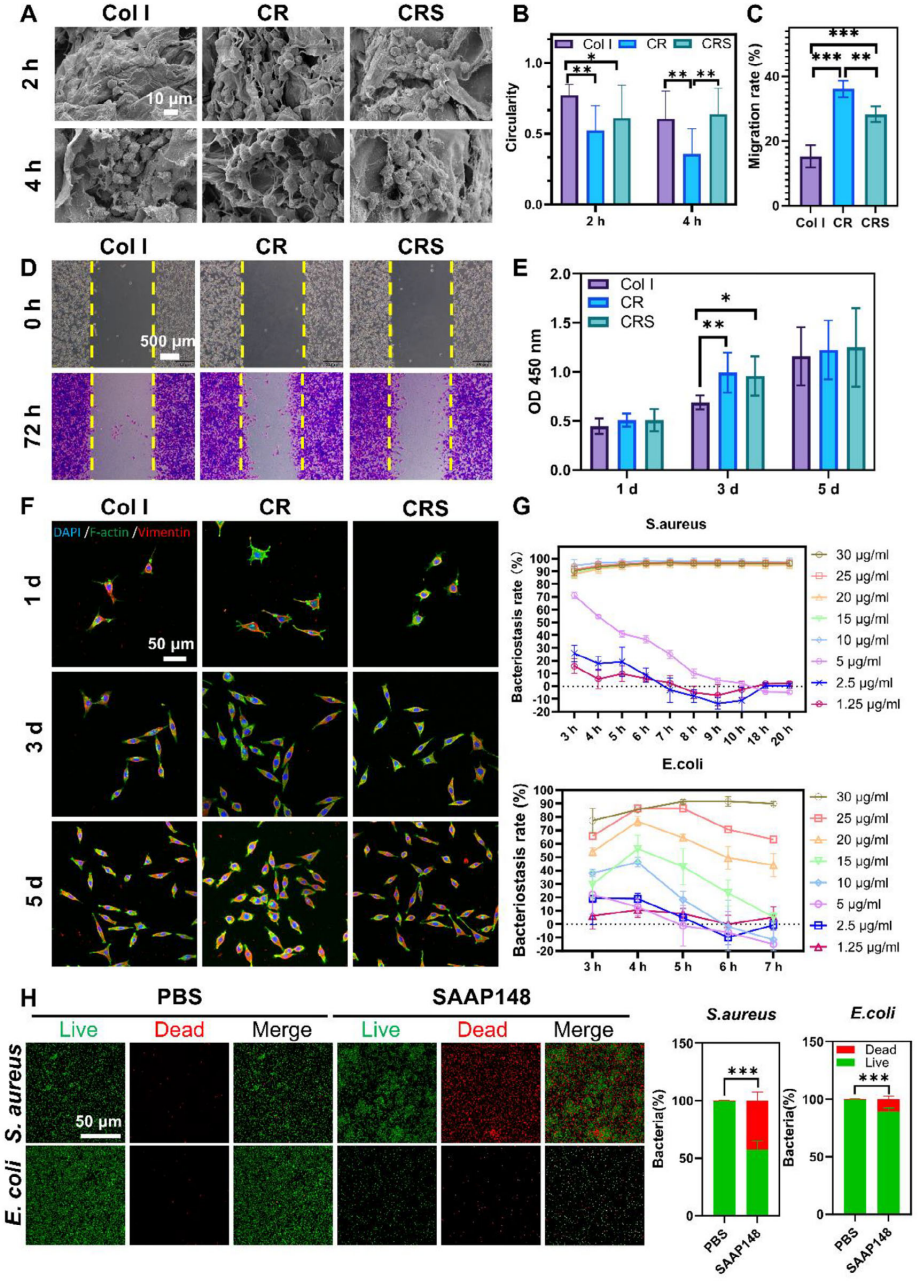
Effect of the Col I, CR, and CRS on fibroblasts and bacteria. After L929 cells were cultured on Col I, CR, and CRS scaffolds for 2 and 4 h, A) cell morphology and B) cell roundness were observed by SEM and analyzed by ImageJ. C,D) L929 migration on the Col I, CR, and CRS scaffolds was evaluated by crystal violet staining and ImageJ. E,F) L929 proliferation on Col I, CR, and CRS scaffolds at day 1, 3, and 5 was observed by immunofluorescence staining and analyzed by CCK8 Kit. G) After CRS loading with SAAP148 (gradient concentration) was co‐cultured with *E. coli* for 7 h or *S. aureus* for 20 h, the growth of bacteria was measured by OD value, H) CRS loaded with SAAP148 was co‐cultured with *E. coli* or *S. aureus* for 20 h, the bacteria were stained by live/dead staining fluorescence, and quantitatively analyzed by ImageJ. Mean ± SD, *p < 0.05, **p < 0.01, ***p < 0.001, *n* = 3.

To evaluate how material surface characteristics influence cellular responses, L929 fibroblast adhesion dynamics were systematically compared across Col I, CR, CRS. Initial cell attachment occurred on all materials within 2 h, but CR surfaces uniquely induced extensive pseudopodal extensions (**Figure** **2**A), with quantitative morphology analysis revealing significant differences (Figure 2B). Col I‐supported cells maintained rounded profiles (circularity = 0.771 ± 0.077) compared to CR (0.522 ± 0.175, ***p < 0.001) and CRS (0.610 ± 0.233, *p < 0.05) (Figure 2B). CR scaffolds further demonstrated accelerated adhesion kinetics, achieving complete cell spreading by 4 h (circularity = 0.637 ± 0.183). This enhanced bioactivity correlates with RADA16‐derived nanofibers (8 – 12 nm diameter observed via SEM), creating a biomimetic nanotopography that promotes integrin α5β1‐mediated focal adhesion complex formation, as verified through actin immunostaining (Figure 2F), which may facilitate mechanotransduction signaling pathways critical for cellular migration and tissue integration. The partial surface coverage of CR by SAAP148 attenuated RADA16‐mediated cellular interactions, however, it resulted in increased cellular roundness and diminished adhesion efficiency on CRS compared to cells cultured on CR (Figure 2B).

#### 2.5.2 Fibroblast Migration on the Col I, CR, and CRS

During wound healing processes involving inflammation, fibrosis, and neovascularization, cellular mechanotransduction pathways mediate shape adaptation and migratory responses. Comparative in vitro scratch assays revealed distinct migration kinetics across scaffold groups (Figure 2C, D; Figure S5, Supporting Information). CR scaffolds demonstrated superior wound closure capacity (36.09 ± 2.59%) compared to Col I controls (15.28 ± 3.43%, ***p < 0.001), while the efficacy of CRS (28.27 ± 2.39%) was slightly reduced compared to CR (**p < 0.01, Figure 2C). This attenuation of CRS correlated with SAAP148 antimicrobial peptide's cationic domains, which potentially interfere with integrin αvβ3‐mediated cell adhesion. Notably, CRS remained significantly elevated over Col I (***p < 0.001, Figure 2C).

#### 2.5.3 Cell Proliferation on the Col I, CR, and CRS

Proliferation analysis via CCK‐8 assays revealed cytocompatibility across all scaffolds, with sustained cell viability (Figure 2E,F; Figure S6, Supporting Information). While initial 24‐h cultures showed comparable cell densities (*p* > 0.05), CR scaffolds demonstrated significant mitogenic enhancement by day 3 (CR vs Col I, **p < 0.01; CRS vs Col I: *p < 0.05). By day 5, confluence‐induced contact inhibition equalized cell numbers across groups (Col I vs CR vs CRS, *p* > 0.05), confirming scaffold biocompatibility.

### 2.6 Antibacterial Properties

The CRS composite demonstrated broad‐spectrum antimicrobial efficacy critical for combating wound infection complications, as quantitatively assessed against Gram‐positive *S. aureus* (ATCC 25 923) and Gram‐negative *E. coli* (ATCC 25 922). Live/dead staining revealed CRS‐loaded SAAP148 (50 µg mL^−1^) induced 42.66 ± 7.53% bacterial mortality in *S. aureus* (***p < 0.001, Figure 2H) and 10.58 ± 2.90% in *E. coli* (***p < 0.001, Figure 2H). Dose‐response profiling identified SAAP148's minimum inhibitory concentration (MIC) at 10 µg mL^−1^ for *S. aureus* with sustained > 90% inhibition over 72 h, while *E. coli* required 30 µg mL^−1^ for equivalent efficacy (Figure 2G). This three‐fold potency difference aligns with SAAP148's net charge, preferentially disrupting Gram‐positive peptidoglycan through electrostatic targeting (Figure 2G,H; Figure S7, Supporting Information).

### 2.7 Hemocompatibility Evaluation

Hemolytic potential of hydrogel eluates (Col I, CR, CRS) was quantified to assess erythrocyte membrane destabilization risks. All materials demonstrated clinically acceptable hemolysis levels below the ISO 10993‐5 safety threshold (5%): Col I (0.72% ± 0.25), CR (0.86% ± 0.66), and CRS (1.58% ± 0.50) (Figure S8, Supporting Information). These statistically indistinguishable values (one‐way ANOVA, *p* > 0.05) confirm minimal hemoglobin release induction, validating blood‐contact safety through preservation of erythrocyte membrane integrity. The observed < 2% hemolysis across groups suggests negligible free radical generation from material degradation byproducts.

### 2.8 Hemostatic Evaluation

**Figure 3:**
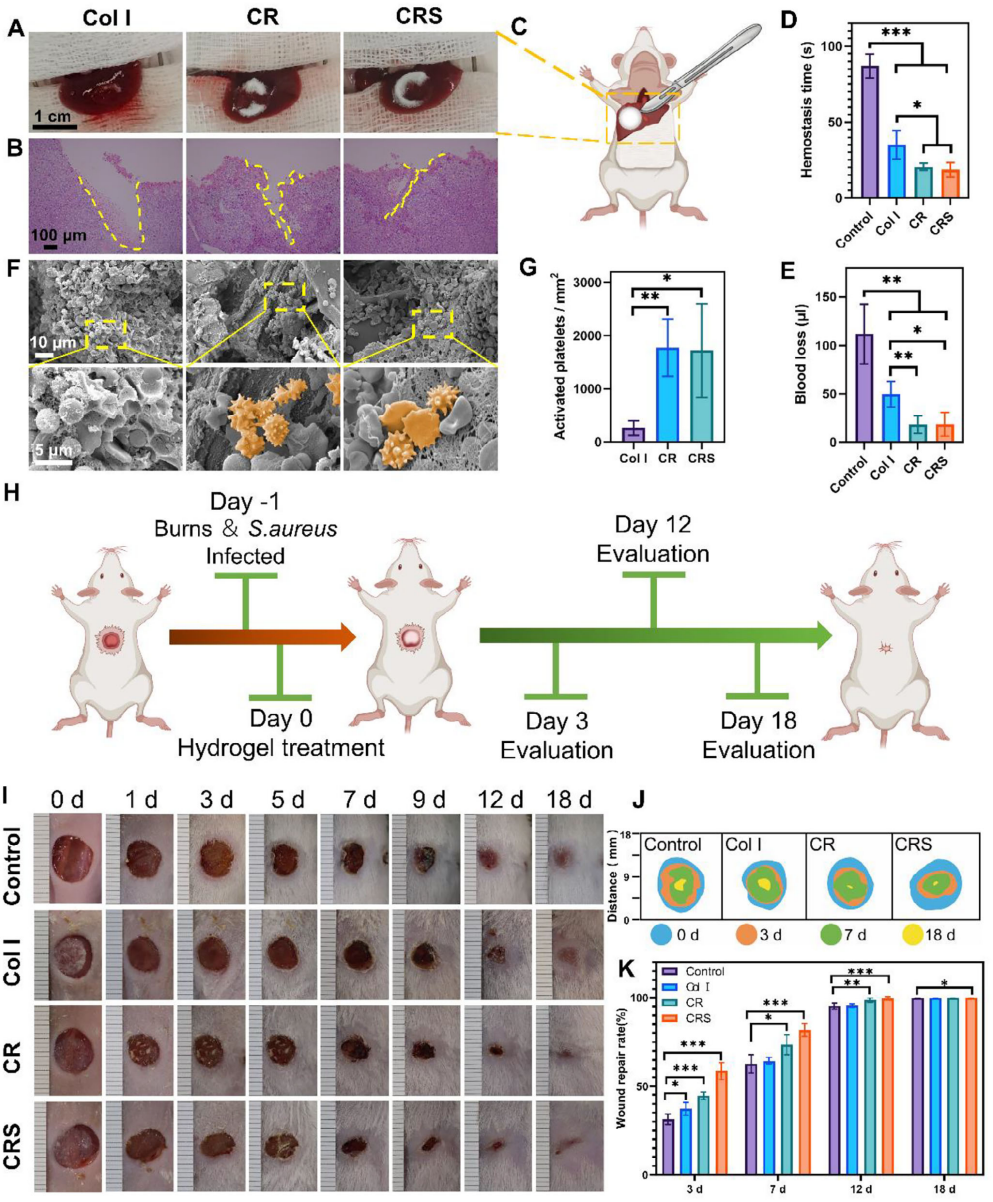
Effect of the Col I, CR, and CRS on hemostasis and infected wound healing. The mouse liver hemostasis model evaluated the hemostasis property of the Col I, CR, and CRS. The hemostatic effect of Col I, CR and CRS on the liver rupture was preliminarily evaluated by observing A) the liver morphology, B) HE staining and C) a schematic representation of hepatic hemostasis. D) Bleeding time and E) bleeding volume of the ruptured liver after using Col I, CR, and CRS. F) SEM images of the Col I, CR and CRS scaffolds after hemostasis experiment in the mouse liver rupture and G) statistical data of activated platelets per square millimeter. The activated platelets are marked with yellow pseudocolor. H) Schematic diagram of the full‐thickness skin injury model. I) The observation of the wounds after treatment on days 1,3, 5, 7, 9, 12, and 18, without treatment as the control group. J) The wound repair rate and K) the area of the wounds were compared among control, Col I, CR and CRS groups. Mean ± SD, *p < 0.05, **p < 0.01, ***p < 0.001, *n* = 3.

The hemostatic efficacy of Col I, CR, and CRS was assessed in a mouse model of left liver lobe injury. A 5 mm incision was created on the exposed liver, and the wound gushed with blood, then each material was immediately applied to the wound until bleeding ceased (**Figure** **3**A,C). Post‐treatment analysis revealed that CR and CRS, leveraging RADA16's viscous nanofiber‐forming properties, minimized wound size compared to Col I (Figure 3B). CR and CRS achieved significantly faster clotting times (21 ± 2 s and 19 ± 5 s, respectively) vs Col I (35 ± 9 s) and gauze (87 ± 8 s; ***p < 0.001 vs gauze; *p < 0.05 vs Col I, Figure 3D), while reducing blood loss by 62% (18.4 ± 9.0 µL and 18.6 ± 12.1 µL) compared to Col I (49.6 ± 13.2 µL, Figure 3E). This enhanced performance stemmed from RADA16's fibrin‐mimetic nanofiber architecture, which accelerated platelet activation and clot stabilization at the injury site (Figure 3F,G). These results demonstrate that CR and CRS offer a statistically superior hemostatic solution for severe hemorrhage, combining rapid clotting, reduced blood loss, and structural mimicry of natural fibrin networks.

### 2.9 Effect on Infected Wound Healing

To assess the antibacterial efficacy and wound repair potential of SAAP 148, CRS was tested in a mouse full‐thickness wound infection model (Figure 3H). After creating wounds and infecting them with bacterial suspension for 24 h, treatments with Col I, CR, or CRS dressings were initiated (Figure 3I). To evaluate the in vivo antibacterial efficacy of the CRS scaffold, wounds on days 1, 3, and 5 post‐treatment were quantitatively analyzed for colony‐forming units (CFUs) (Figure S9, Supporting Information). The collagen sponge covering the wound surface effectively isolated most bacteria from direct contact with the wound, resulting in a statistically significant reduction in bacterial loads in the Col I, CR, and CRS groups compared to the blank control group (***p < 0.001, Figure S9A,B, Supporting Information). The wound surface gradually scabbed over by days 3‐5, and the bacterial load in the blank control group also decreased. However, the bacterial load of wounds in the Col I, CR, and CRS groups was still significantly lower than that in the control group (***p < 0.001, Figure S9C,D, Supporting Information). In particular, the CRS scaffold was effective in inhibiting bacterial colonization in vivo, and the bacterial load was significantly lower than that of the Col I (Day 1: ***p < 0.001, Day 3: **p < 0.01, Day 5: *p < 0.05) and CR groups (Day 1 and 3: **p < 0.01, Day 5: *p < 0.05, Figure S9B–D, Supporting Information).

**Figure 4:**
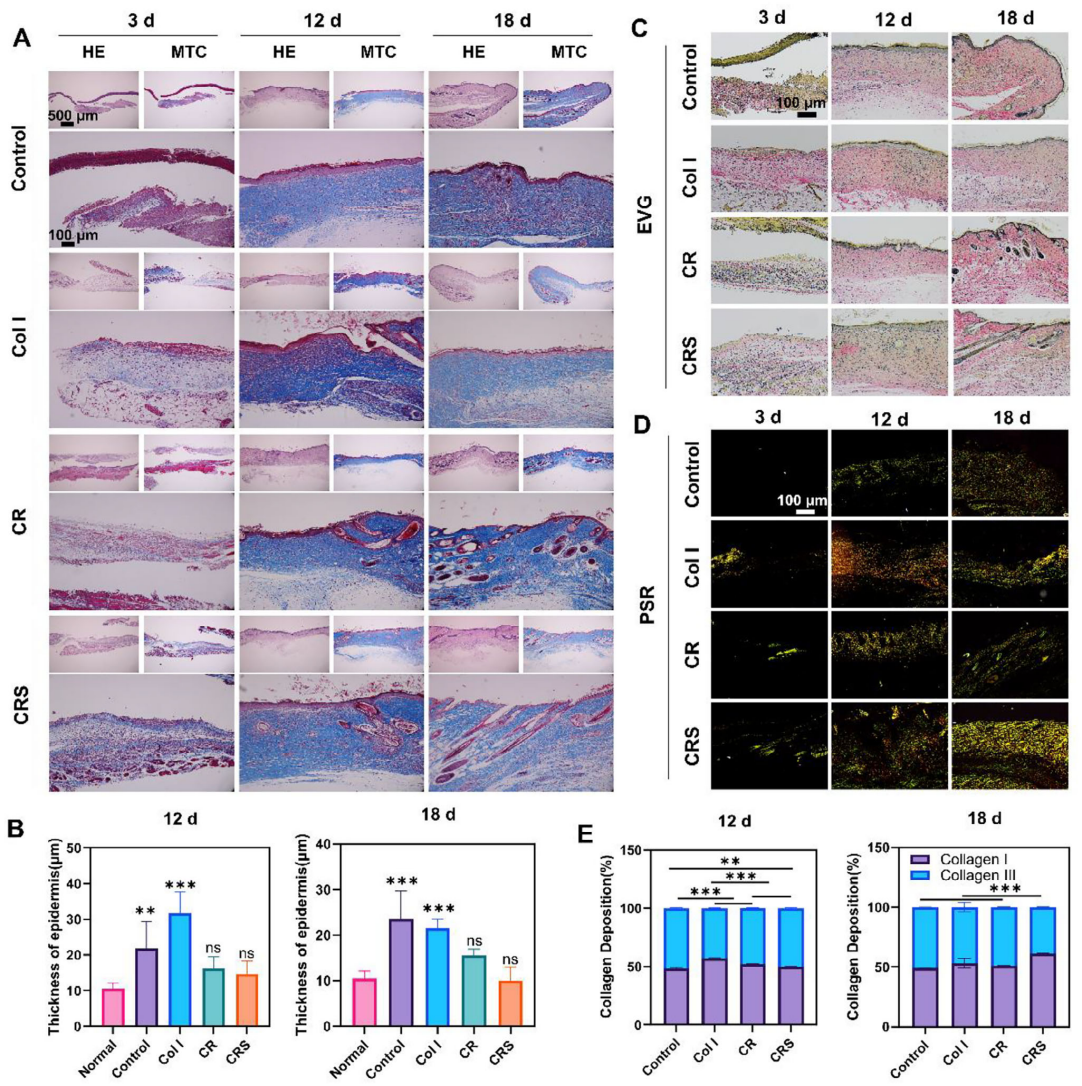
Newly formed skin in infection wound model evaluated by histological staining. A) Col I, CR, and CRS scaffolds were used for treatment on infected wounds and were observed by HE and Masson staining on post‐operative days 3, 12, and 18. No treatment as the control group. B) Skin thickness measurements at postoperative days 12 and 18. C) EVG staining of tissues at 3, 12, and 18 days after healing. D) PSR stained tissue sections at postoperative days 3, 12, and 18. E) Type I/III collagen ratio analysis by PSR staining at 12 and 18 days after surgery. Mean ± SD, **p < 0.01, ***p < 0.001, *n* = 3.

CRS demonstrated superior performance, significantly reducing wound area at all postoperative time points compared to the control group (***p < 0.001 on days 3, 7, and 12; *p < 0.05 on day 18), outperforming both Col I and CR (Figure 3I–K). Histological analysis via H&E and MTC further confirmed CRS enhanced the infected wound healing (**Figure** **4**A). By day 3, the control group exhibited only blood crust formation, while Col I‐treated wounds showed blue‐stained collagen deposition (Figure 4A). In contrast, CR and CRS groups displayed fibrous tissue formation alongside collagen (Figure 4A). By day 12, all treatment groups exhibited collagen deposition at the wound site, with Col I, CR, and CRS showing markedly greater collagen accumulation than the control (Figure 4A). These findings highlight CRS's dual functionality in combating infection and accelerating tissue regeneration, positioning it as a promising therapeutic candidate for infected wound management.

Notably, hair follicle formation was observed in the newly regenerated skin of both CR and CRS groups by day 18 post‐treatment, with CRS exhibiting a significantly higher density of hair follicles compared to CR (Figure 4A). While macroscopic examination indicated complete wound closure across all groups by day 18, histological analysis revealed critical differences in tissue architecture. In the control group, collagen fibers within the dermis remained disorganized with excessive deposition, and no hair follicles were detected in the regenerated skin. Similarly, the Col I group showed minimal hair follicle formation. In contrast, CR and CRS groups demonstrated robust hair follicle regeneration, closely resembling native skin morphology (Figure 4A). Additionally, epidermal thickness, which is a marker of restored skin integrity progressively normalized during later healing stages. On day 12, the control group (21.81 ± 7.537 µm) and Col I group (31.75 ± 5.867 µm) exhibited significantly thicker epidermis compared to normal skin (10.49 ± 1.634 µm; ***p < 0.001 and **p < 0.01, respectively), whereas CR (16.29 ± 3.240 µm) and CRS (14.66 ± 3.557 µm) groups showed no statistical difference from normal skin (Figure 4B). By day 18, epidermal thickness in the CR group (15.59 ± 1.314 µm) remained comparable to normal skin, while the CRS group achieved near‐complete restoration (10.04 ± 3.009 µm vs normal 10.49 ± 1.634 µm) (Figure 4B). These results underscore CRS's superior capacity to promote structurally and functionally mature skin regeneration, characterized by organized collagen deposition, hair follicle neogenesis, and physiological epidermal thickness.

EVG staining, which effectively distinguishes elastic fibers (essential for skin elasticity and resilience) from collagen fibers, was employed to evaluate dermal structural recovery and collagen organization during wound healing, which was a critical factor in mitigating hypertrophic scarring caused by excessive collagen deposition. By day 3 post‐injury, no continuous epidermal layer had formed in any group, while by day 12, all groups exhibited initial collagen deposition and elastic fiber regeneration, albeit with disorganized arrangements. Strikingly, by day 18, the CRS group demonstrated superior dermal restoration, characterized by tightly aligned collagen bundles and neatly distributed elastic fibers that closely resembled native skin architecture. In contrast, control and other treatment groups still exhibited fragmented collagen networks and irregular elastic fiber patterns, indicating incomplete healing (Figure 4A,C). These results underscore CRS's unique ability to orchestrate structurally organized tissue regeneration, simultaneously restoring functional elastic fiber networks (Figure 4C). To further characterize collagen maturation, Sirius red staining was employed to differentiate type I collagen (yellow/orange) from type III (green) (Figure 4D). It was important that the Col I materials should be distinguished from the Col I in the skin though the histological morphology, which was calculated for Col I in the newly formed skin in this experiment. Early healing stages (day 12) featured predominant type III collagen, which supports provisional matrix formation, while later stages (day 18) saw increased type I collagen, essential for mechanical strength. Quantitatively, the CRS group exhibited accelerated collagen deposition by day 12 and a significantly higher type I/III collagen ratio by day 18 (***p < 0.001, Figure 4E), indicating advanced maturation. Combined with histological evidence of organized dermal repair and elastic fiber restoration, these findings demonstrate that CRS uniquely drives both structural and compositional skin regeneration, transitioning collagen to its mature, strength‐enhancing form.

### 2.10 Analysis of the Mechanism by which CRS Promotes Healing of Infected Wounds

**Figure 5:**
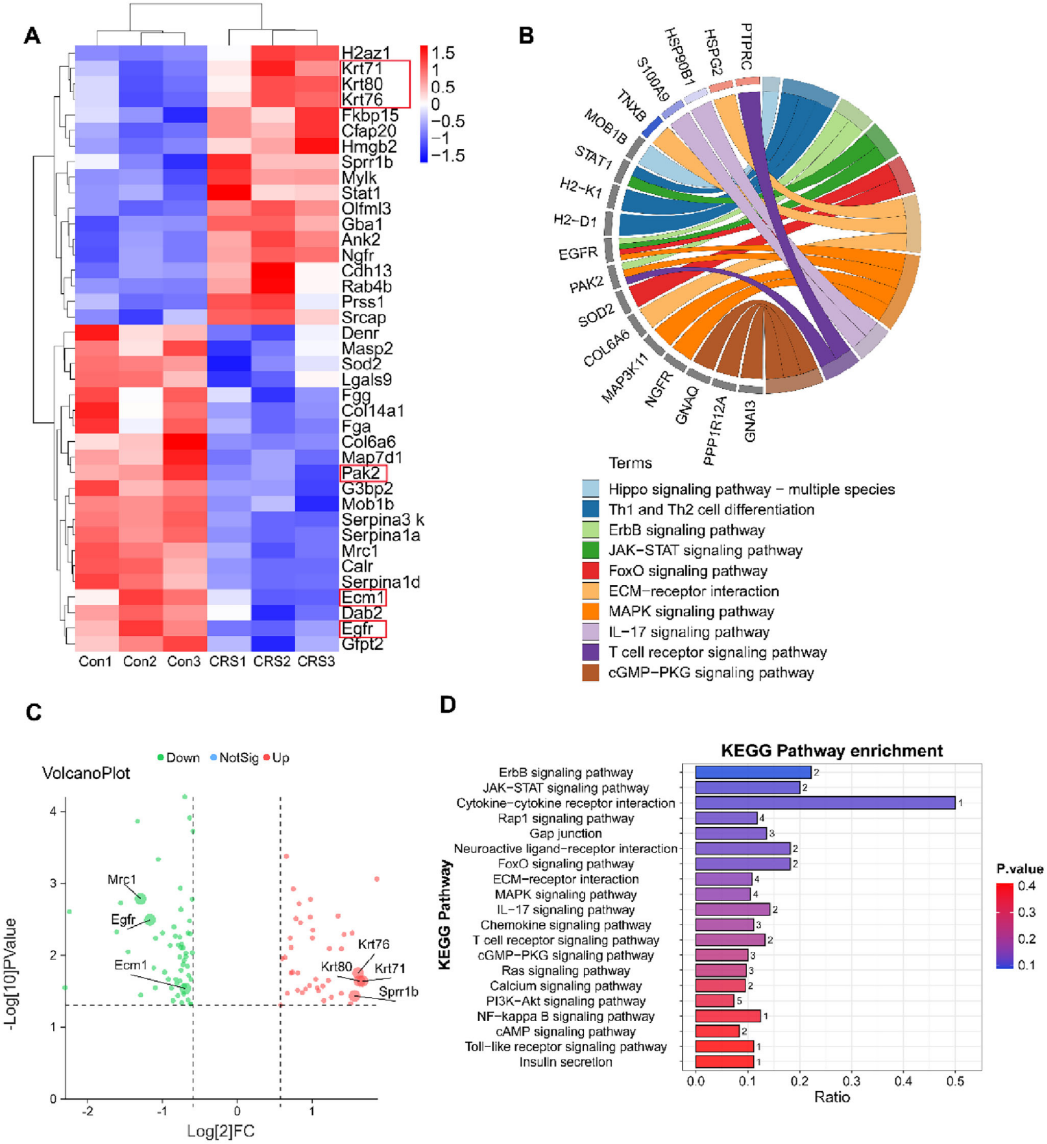
Spatial proteomics sequencing. On post‐operative day 18, tissues were harvested from both the CRS‐treated and control groups for spatial proteomic profiling. Subsequent analyses included: A) Heatmap of differential proteins, B) KEGG chord diagram visualizing pathway‐protein associations. C) Volcano plot of differential proteins, D) KEGG pathway enrichment bar graph.

**Figure 6:**
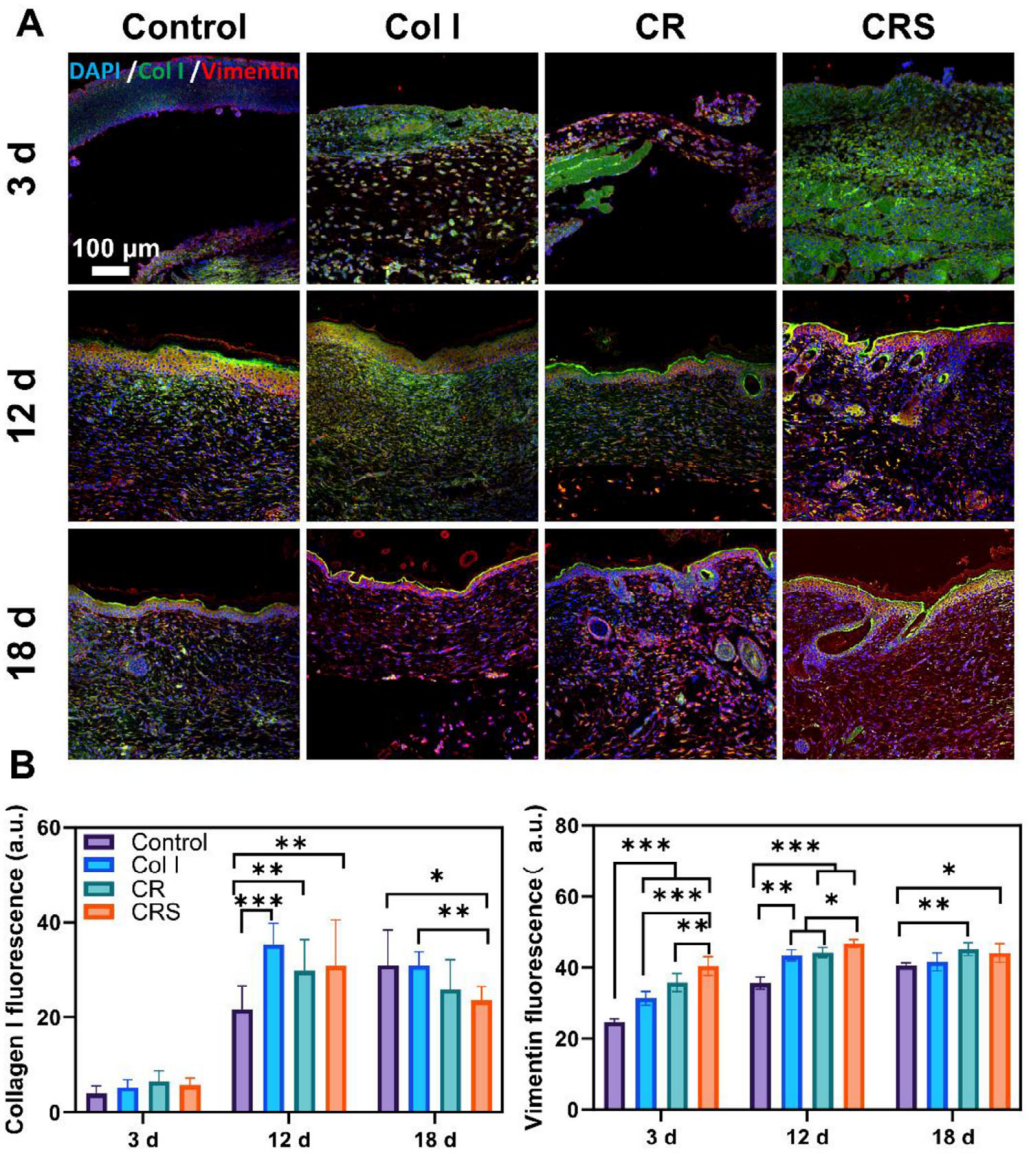
Fluorescent labeling of type I collagen and fibroblasts in the newly formed skin. A) Immunofluorescence visualization of Col I deposition (green) and vimentin‐labeled fibroblasts (red) at post‐wounding days 3, 12, and 18. B) Statistical analysis of the fluorescence intensity of Col I and vimentin in control, Col I, CR, and CRS groups. Mean ± SD, *p < 0.05, **p < 0.01, ***p < 0.001, *n* = 3.

**Figure 7:**
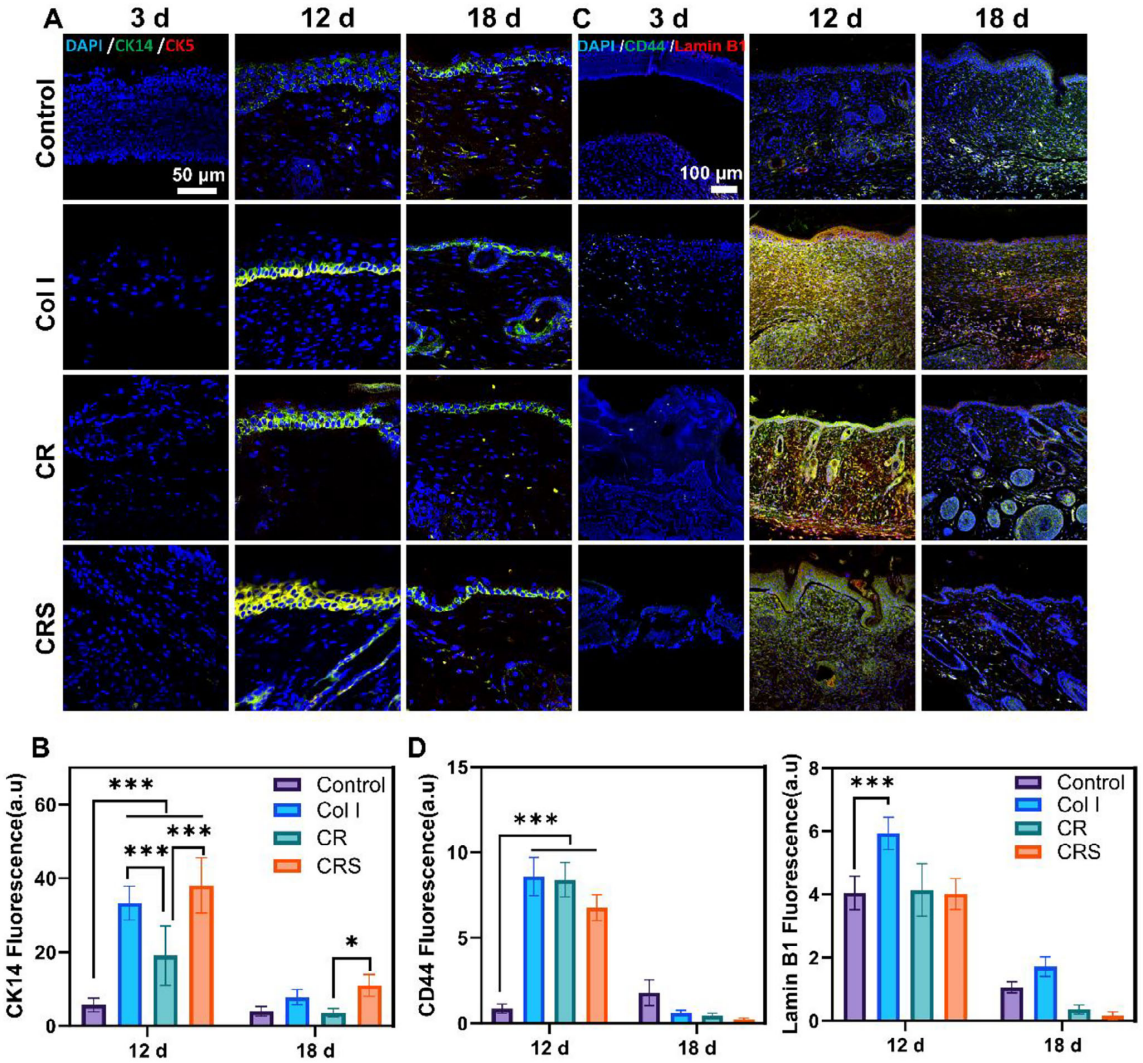
Fluorescent staining of keratinocytes, CD44 and LaminB in mouse newly formed skin. A) CK14 (green) and CK5 (red) co‐label keratinocytes in the epidermis of newly formed skin. B) Statistical analysis of the fluorescence intensity of CK14 in control, Col I, CR, and CRS groups on post‐operative days 12 and 18. C) CD44 (green) and Lamin B1 (red) co‐label newly formed skin. D) Statistical analysis of the fluorescence intensity of CD44 and Lamin B1 in control, Col I, CR, and CRS groups on post‐operative day 12 and 18. Mean ± SD, ***p < 0.001, *n* = 3.

**Figure 8:**
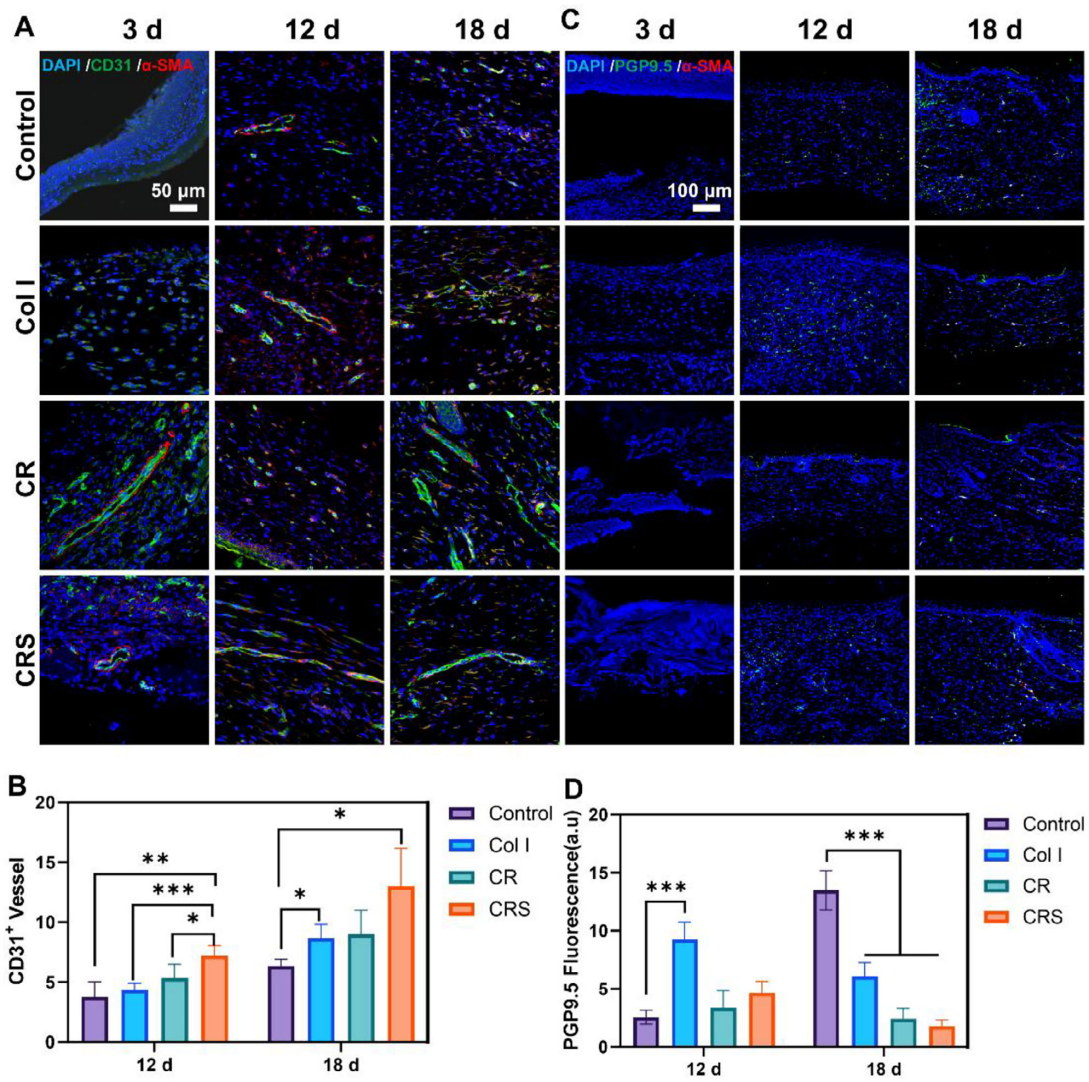
Fluorescence staining of blood vessels and nerve fibers in mouse newly formed skin. A,B) CD31 (green)‐labeled endothelial cells (ECs) and α‐SMA (red)‐labeled smooth muscle cells demonstrated co‐localized vascularization. Quantitative assessment of CD31‐positive neovascular structures was conducted on day 12 and day 18 tissue specimens. C, D) PGP9.5 (green) and α‐SMA (red) immunostaining revealed nerve fibers in newly formed tissue, with fluorescence intensity quantification of PGP9.5‐positive neural components performed in corresponding day 12 and day 18 regenerated tissues. Mean ± SD, *p < 0.05, **p < 0.01, ***p < 0.001, *n* = 3.

Spatial proteomic sequencing of the Control and CRS groups at 18 days post‐injury identified significant differential protein expression in CRS‐treated skin, including enrichment of ERBB/MAPK, Ras, PI3K‐AKT signaling pathway, etc. (**Figure** **5**B,D), upregulated markers (Keratin: Krt76, Krt 71, Krt80, and Sprr 1b) and downregulated markers (Egfr, Ecm1, Mrc1) linked to skin tissue repair (Figure 5A,C; Figure S10, Supporting Information). Immunofluorescence staining further revealed enhanced fibroblast activity (Vimentin^+^) and collagen I deposition in CRS‐treated tissues compared to controls (*p < 0.05, **Figure** **6**A,B), corroborating its superior dermal regeneration. Epidermal repair dynamics, tracked via keratin expression (CK14, CK5), demonstrated that CRS accelerated keratinocyte differentiation and restored normal epidermal thickness by day 18, whereas the Control and Col I groups lagged with persistent hyperplasia (**Figures** **7**A,B; S11, Supporting Information). CRS also facilitated early‐stage re‐epithelialization through upregulated CD44 (critical for keratinocyte migration) and Lamin B1 (supporting nuclear integrity during rapid proliferation), with both proteins returning to baseline levels post‐healing (Figure 7C,D). Newly formed vessels, assessed via CD31 (endothelial marker), were markedly enhanced in CRS‐treated wounds, with significantly higher CD31^+^ vessel counts at day 12 versus Control (***p < 0.001), Col I (***p < 0.001), and CR (*p < 0.05) groups (**Figure** **8**A,B). Nerve fiber regeneration paralleled vascular growth, peaking earlier in CRS and CR groups before pruning as repair concluded, while Control and Col I groups exhibited delayed nerve dynamics (Figure 8C,D).

**Figure 9:**
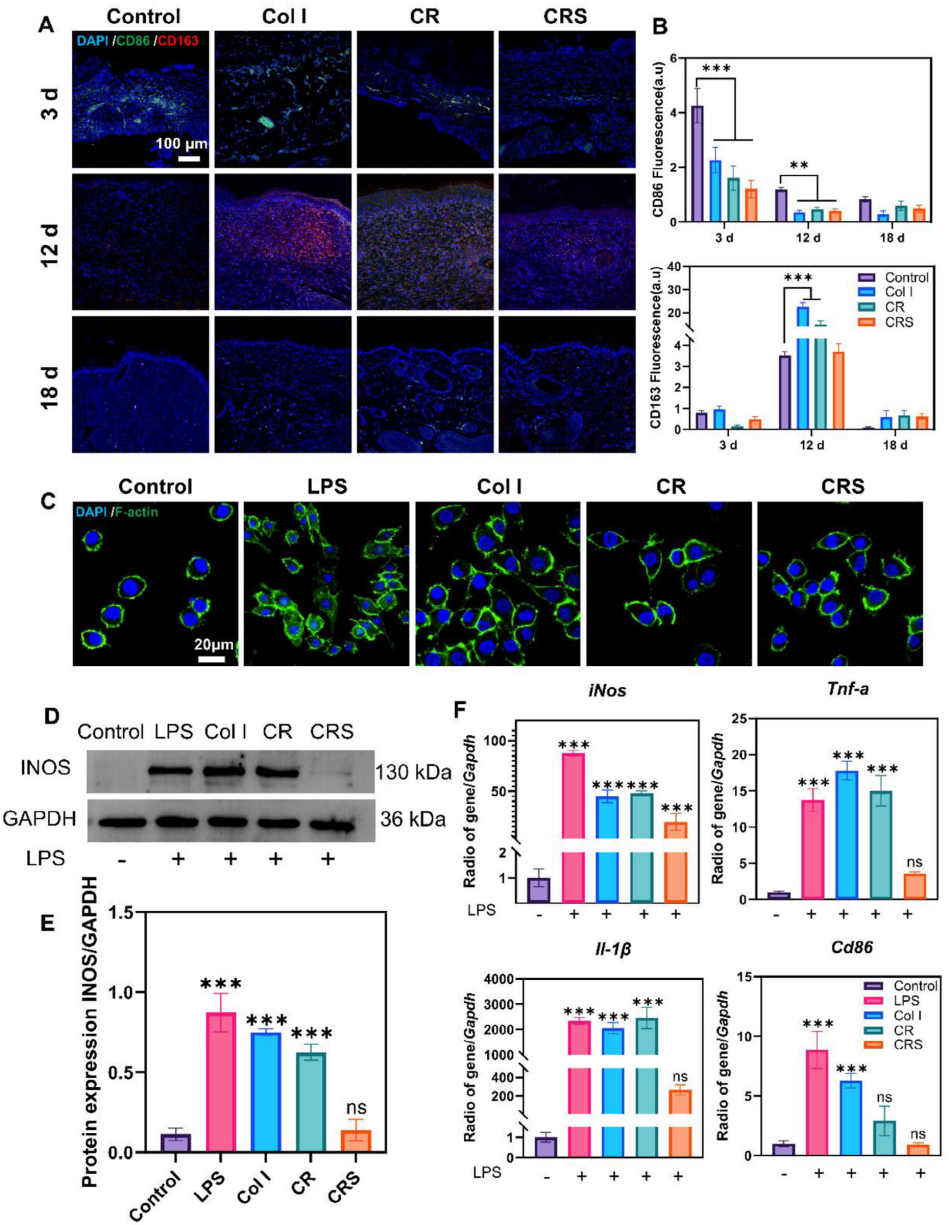
Effect of the Col I, CR, and CRS on macrophage polarization. A) Newly formed tissues from Col I, CR and CRS‐treated groups on post‐operative days 3, 12, and 18 were subjected to immunofluorescence staining for M1 macrophages (CD86^+^, green) and M2 macrophages (CD163^+^, red). B) Quantitative analysis of CD86 and CD163 fluorescence intensities. C) CL488‐Phalloidin labeling of RAW264.7 cells cultured in different treatment groups for 6 h. D, E) WB analysis of iNOS protein expression with grayscale intensity quantification in RAW264.7 cells after 6 h culture across treatment groups. F) RT‐qPCR detection of M1 phenotype‐associated gene expression in RAW264.7 cells following 5 h culture under various treatments. Mean ± SD, **p < 0.01, ***p < 0.001, *n* = 3.

**Figure 10:**
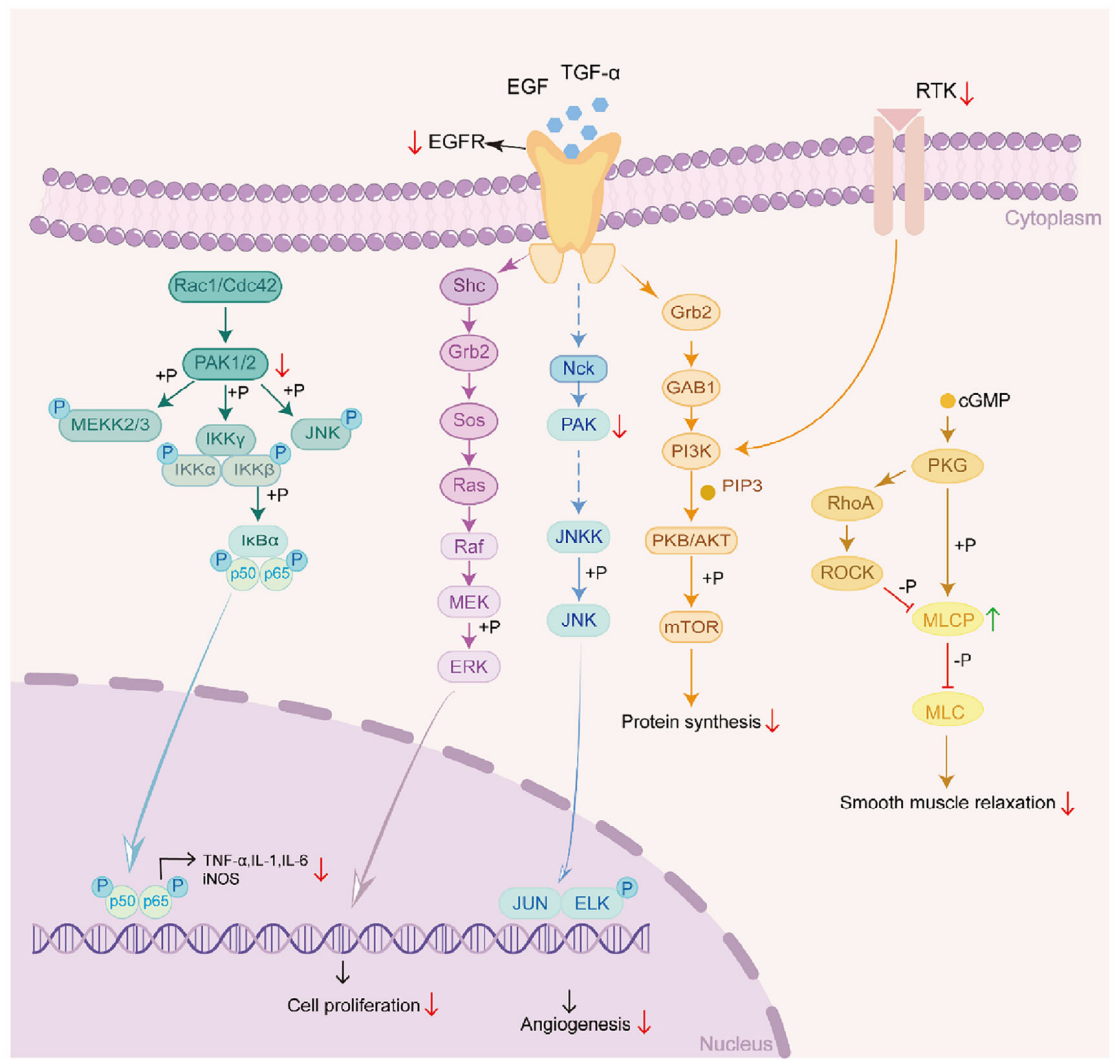
The CRS group is related to the regulation of cell activity related signaling pathways. Based on spatial proteomics analysis of wound healing tissue samples (volcano plots and KEGG pathway enrichment), the key signaling pathways regulating repair processes were mapped. In the CRS group, EGFR downregulated cell proliferation‐related activities via the ERBB/MAPK signaling pathway; RTK suppressed protein synthesis through the mTOR/PI3K‐Akt signaling axis; and PAK attenuated inflammatory responses by inhibiting the expression of pro‐inflammatory mediators (e.g., IL‐1β, TNF‐α) via the JNK/p38 MAPK cascade.

In the early stages of wound healing, M1 macrophages initiate inflammation by releasing pro‐inflammatory cytokines (e.g., TNF‐α, IL‐1β), while prolonged M1 activation can hinder repair. Conversely, M2 macrophages emerge later to promote tissue remodeling via anti‐inflammatory cytokines and growth factors. On day 3 post‐injury, all groups exhibited inflammatory responses, with the Control group showing the highest M1 marker CD86 expression (***p < 0.001) and minimal M2 activity (**Figure** **9**A,B). By day 12, the Control group still displayed significant inflammation (**p <0.01) alongside elevated M2 marker CD163 (***p < 0.001), while Col I and CR groups showed increased M2 macrophages (***p < 0.001). Notably, the CRS group accelerated tissue repair which correlated with reduced M2 marker expression, suggesting earlier resolution of the repair phase. By day 18, macrophage activity declined across all groups, indicating the nearing completion of healing (Figure 9A,B). After bacterial infection, the released LPS acted as a nonspecific immunogen, and when entering the microcirculation interacted with host effector cells (mainly monocytes, macrophages, and neutrophils) to secrete biologically active molecules, such as tumor necrosis factor‐alpha (TNF‐α), interleukin 1 (IL‐1), interleukin 2 (IL‐2), and interleukin 6 (lL‐6), causing increased inflammation and other clinical syndromes. To validate these findings, Raw264.7 cells were treated with Col I, CR, or CRS during LPS‐induced M1 polarization. While Col I and CR groups showed significant M1 morphology (similar to LPS controls), CRS group markedly suppressed M1 polarization, with fewer cells adopting the pro‐inflammatory phenotype (Figure 9C). WB and qPCR confirmed the anti‐inflammatory effects of CRS, which may be due to the neutralization of LPS by SAAP148. INOS protein (***p < 0.001, Figure 9D,E) and M1‐associated genes (*iNOS*, *TNF‐α*, *CD86*, *IL‐1β*; ***p < 0.001, Figure 9F) were significantly downregulated. qPCR of healed tissues further revealed sustained *Egf* upregulation in the CRS group at days 12 and 18 (***p < 0.001, Figure S12, Supporting Information), alongside a rapid decline in inflammatory factors (*TNF‐α, IL‐1β, Arg1*) after day 7, underscoring the dual role of CRS in prolonging regenerative signaling while curtailing inflammation. The above results align with the differential protein expression levels detected via spatial proteomics sequencing, including the downregulation of inflammatory mediators such as TNF‐α and IL‐1β (Figures 5 and 10).

Collectively, CRS accelerates wound healing through a synergistic integration of structural repair, cellular regulation, and immune modulation. By restoring near‐native dermal architecture via organized collagen deposition and elastic fiber regeneration (Figure 4C,D), CRS minimizes scarring while enhancing mechanical strength through fibroblast activation (Vimentin^+^) and collagen I maturation. Rapid epidermal normalization, driven by accelerated keratinocyte differentiation (Figures 4B and 7A), is complemented by early neovascularization (CD31^+^ vessels, Figure 8A,B) and synchronized nerve regeneration, ensuring nutrient delivery and sensory recovery (Figure 8C,D).

CRS dynamically balances macrophage responses, suppressing pro‐inflammatory M1 activity (reduced CD86, iNOS, TNF‐α; ***p < 0.001, **Figure** **9**A–F) during initial inflammation while preventing prolonged M2 dependency, thereby streamlining the transition to tissue remodeling. SAAP148 within CRS further amplifies anti‐inflammatory effects (Figure 9C–F), sustains regenerative signaling via elevated *Egf* (Figure S12, Supporting Information), and accelerates post‐day 7 resolution of inflammatory cytokines (*TNF‐α, IL‐1β*). At post‐day 18, spatial proteomic sequencing revealed significant downregulation of EGFR, RTK, and PAK protein expression in the CRS group compared to controls (Figure 5B–D). Wound healing‐related signaling pathways were mapped based on KEGG analysis obtained by protein sequencing in the control and CRS groups at 18 days postoperatively (**Figure** **10**). EGFR regulates cell proliferation, angiogenesis, and protein synthesis through Ras/ERK, PAK/JNK, or PI3K/AKT signaling pathways. Reduced PAK1/2 levels suppressed inflammatory cytokines (TNF‐α, IL‐1, IL‐6) via the IκBα/p50/p65 axis. These coordinated mechanisms establish CRS as a transformative therapy for scar‐free, functionally mature wound repair in both acute and chronic contexts.

## 3 Discussion

The repair of skin wounds is a dynamic, multi‐stage process encompassing hemostasis, inflammation, proliferation, and remodeling. While this cascade is evolutionarily conserved, its dysregulation in infected or chronic wounds often leads to impaired healing characterized by persistent inflammation, microbial colonization, and aberrant extracellular matrix (ECM) deposition. Our study introduces CRS, a multifunctional composite scaffold engineered to synchronize these phases through biomimetic design and bioactive modulation. Unlike conventional single‐function dressings, CRS uniquely integrates fibrin‐mimetic hemostasis, antimicrobial defense, and ECM‐mimicking nanostructures to address the interconnected challenges of wound repair. The hemostatic superiority of CRS stems from RADA16's ability to recapitulate fibrin's nanofibrous architecture, which not only accelerates clotting (19 ± 5 s, 18.6 ± 12.1 µL vs 35 ± 9 s, 49.6 ± 13.2 µL for Col I; *p < 0.05, Figure 3D,E) but also establishes a provisional matrix conducive to cell infiltration (Figure 2C). This structural mimicry outperforms chitosan/ε‐polylysine hydrogels (73.96 µL), ^[^ ^24^ ^]^ commercial gelatin sponges (28 ± 5 s and 34.7 ± 5.3 µL blood loss)^[^ ^25^ ^]^ and commercial collagen sponge (39 ± 3 s and 49.8 ± 9.4 µL blood loss, Figure S13, Supporting Information). This enhanced efficacy arises from the dual mechanism of RADA16:i) physical entrapment of blood components, mimicking SURGICEL barrier function,^[^ ^26^ ^]^ and ii) active platelet through its nanofibrous architecture (Figures 1D and 3F,G; Figure S3, Supporting Information). Unlike passive cellulose‐based absorbers that merely trap blood, RADA16 proactively stimulates thrombus formation. Furthermore, in contrast to calcium‐alginate hemostats known to induce inflammation,^[^ ^27^ ^]^ RADA16 undergoes degradation into non‐immunogenic amino acids,^[^ ^28^ ^]^ eliminating risks of immune rejection.

Beyond hemostasis, CRS addresses the inflammation through SAAP148, an antimicrobial peptide that disrupts bacterial membranes via non‐receptor‐mediated mechanisms.^[^ ^18^ ^]^ Previous studies have exemplified the critical role of collagen for LL37 to function. First, collagen achieves ultra‐high loading (> 240 µm) of LL37 through the specific binding domain (CBD), its 3D fiber network forming a physical barrier to eliminate the cytotoxicity brought by LL37. Critically, collagen's natural slow‐release property successfully prolonged the antimicrobial activity of LL37.^[^ ^29^ ^]^ This load‐barrier‐slow‐release trinity makes collagen an ideal platform for solving the dilemma between toxicity, stability and long‐lasting delivery of antimicrobial peptides. Due to stronger antimicrobial activity than direct loading of LL37, the LL37‐derived peptide SAAP148^[^ ^18^ ^]^ was chosen as the antimicrobial peptides (AMPs) in the wound dressing of our study. At 10 µg mL^−1^, SAAP148 achieves over 90% inhibition of *S. aureus* (Figure 2G), which is a potency surpassing gentamicin and rifampicin at equivalent doses, while avoiding antibiotic resistance.^[^ ^30^ ^]^ The CRS scaffold delivered SAAP148 via a controlled release profile (15 – 18% at 24 h; 23 – 37% at 72 h, Figure 1N), contrasting with SAAP148‐release nanogels (37 – 41% within 72 h) reported by Gent et al.^[^ ^31^ ^]^ This Col + RADA16 modulated delivery can also avoid sub‐inhibitory concentrations (< MIC) and resistance development of other drugs. Notably, CRS achieved 90% *E. coli* inhibition at 30 µg mL^−1^ (Figure 2G), outperforming silver nanoparticle dressings requiring > 50 µg mL^−1^ with cytotoxic side effects.^[^ ^32^ ^]^ The histology staining showed that CRS treatment did not cause toxicity to the heart, liver, spleen, lung, or kidney (Figure S14, Supporting Information). The CRS scaffolds synergistically combine RADA16‐driven hemostasis and SAAP148‐mediated antimicrobial activity with pro‐regenerative cellular effects. Notably, CR and CRS significantly enhanced fibroblast adhesion after 2 h of culture, as evidenced by reduced L929 circularity (CR: 0.522 ± 0.175, **p < 0.01; CRS: 0.610 ± 0.233, *p < 0.05) compared to Col I controls (0.771 ± 0.077, Figure 2B). This improvement surpasses natural ECM hydrogels (circularity ≈ 0.8),^[^ ^33^ ^]^ suggesting the nanofibrous architecture of RADA16 mimics native ECM topology and promote integrin‐mediated adhesion. Cell migration assays revealed CRS's superior chemotactic potential, achieving 28.27 ± 2.39% wound closure versus 15.28 ± 3.43% for Col I group (***p < 0.001, Figure 2C,D). While slightly lower than VEGF‐loaded gelatin methacryloyl,^[^ ^34^ ^]^ CRS accomplishes this without exogenous growth factors, which is a critical advantage given the regulatory hurdles of cytokine therapies.^[^ ^35^ ^]^ Comparatively, decellularized dermal matrix (DDM) shows a similar migration of CR (36.09 ± 2.59%) or CRS (28.27 ± 2.39%),^[^ ^36^, ^37^ ^]^ indicating that CRS's nanofiber design rivals biologically derived scaffolds through synthetic simplicity. Proliferation rates further highlight the regenerative capacity of CRS. After 72 h, CR (**p < 0.01) and CRS (*p < 0.05) significantly increased cell density compared to the Col I group (Figure 2E,F). This aligns with findings that aligned nanofibers promote cellular bridge formation, thereby enhancing stem cell mechanotransduction.^[^ ^38^ ^]^ Crucially, CRS maintains bioactivity without cytotoxic cross‐linkers, addressing a key limitation in clinical translation.

The translational efficacy of CRS was validated in full‐thickness murine wound models, where it demonstrated superior regenerative performance compared to both Col I and CR. CRS‐treated wounds achieved 60% closure within 3 days, surpassing commercial hydrogel dressings, such as HyStem,^[^ ^39^ ^]^ and approaching the efficacy of stem cell‐laden scaffolds^[^ ^40^ ^]^ without requiring living components or biological factors. By day 12, CRS attained over 98% epithelialization (Figure 3H–K), outperforming decellularized amniotic membranes (≈50% at 14 days)^[^ ^41^, ^42^ ^]^ and matching autograft outcomes.^[^ ^43^ ^]^ Histologically, CRS induced dermal‐epidermal maturation indistinguishable from unwounded skin by day 18, featuring parallel collagen bundles (MTC staining, Figure 4A) and physiological epidermal stratification (Figures 6A and 7A), which is a stark contrast to the disorganized fibers in Col I/CR groups and hyperproliferative epidermis seen in silver dressing‐treated wounds.^[^ ^44^, ^45^ ^]^ The effect of CRS on suppressing excessive fibrosis can be further analyzed by measuring the ratio of Type I to Type III collagen. The experimental results showed that 18 days after injury repair, the type I/III collagen ratio in the CRS group (1.59) was significantly higher than that in the control group (0.97, ***p < 0.001, Figure 4E). The ratio approaching the ideal 2:1 proportion^[^ ^46^ ^]^ endows the collagen network with both the mechanical strength of type I collagen and the elasticity of type III collagen, promoting polarized extension of fibroblasts. In the intermediate stage of repair (12 days, 42.12% higher than the control group), the CRS scaffold upregulated type I collagen, achieved microenvironment remodeling through mechanical protection, and accelerated the healing of skin damage repair (Figure 6B). At the end of the damage repair (18 days, 23.95% lower than the control group), the remodeling of the extracellular matrix was basically complete, and the appropriate reduction of type I collagen was beneficial to inhibiting the formation of scars caused by excessive fibrosis (Figure 6B).

This structured regeneration mirrors the “scarless healing” paradigm,^[^ ^47^ ^]^ which is realized by CRS through topographical guidance by RADA16, anti‐inflammatory regulation by SAAP148^[^ ^21^ ^]^ (Figure 9). Spatial proteomic sequencing of the Control and CRS groups at 18 days post‐injury identified significant differential protein expression in CRS‐treated skin, including ERBB/MAPK, Ras, PI3K‐AKT signaling pathway etc. (Figures 5B,D and 10). Some of the signaling pathways involved in skin damage repair process, such as PI3K‐Akt signaling pathway can accelerate hair follicle regeneration by promoting hair follicle stem cells (HFSCs) differentiation and epithelial‐mesenchymal transition (EMT) process to accelerate hair follicle regeneration and promote wound healing,^[^ ^48^ ^]^ and the down‐regulation of RAS/p38 MAPK/NF‐κB signaling pathway can inhibit inflammatory response and promote cell proliferation.^[^ ^49^ ^]^ Significant differences in protein expression between the control and CRS groups also included some upregulated markers (Keratin: Krt76, Krt71, Krt80, and Sprr1b), and downregulated markers (Egfr, Ecm1, and Mrc1) linked to skin tissue repair (Figure 5A,C; Figure S10, Supporting Information). Among them, the up‐regulation of CK14 in the keratin family by CRS‐induced expression was verified (Figure 7A,B), and CK14 has been reported to contribute to re‐epithelialization during wound healing.^[^ ^23^ ^]^ For example, CK14 contributes to the maintenance of cell proliferative potential and can increase AKT phosphorylation, and the PI3K‐AKT signaling pathway regulates cell metabolism and growth during keratinocyte differentiation, promotes keratinocyte differentiation, facilitates cell proliferation and inhibits spontaneous keratinocyte differentiation.^[^ ^50^, ^51^, ^52^ ^]^ While the p38/MAPK signaling pathway can integrate and mediate various signals (eg. EGFR) involved in keratinocyte differentiation, when the p38/MAPK signaling pathway is inhibited, the expression of proteins such as CK5, CK14, and p‐Akt is simultaneously downregulated. Collectively, these findings validate the triphasic action of CRS: i) sub‐20s hemostasis; ii) sustained antimicrobial protection (10 µg mL^−1^ MIC against *S. aureus* and 30 µg mL^−1^ against *E. coli*), and anti‐inflammatory effect; iii) matrix remodeling results exceeding current ECM and collagen matrices.

## 4 Conclusion

This work establishes a strategy for infected wound repair that orchestrates hemostasis, antimicrobial defense, and scarless regeneration via mechanobiological programming and immunomodulation. The CRS hydrogel, engineered through hierarchical integration of the microporous architecture of Col I, RADA16's fibrin‐mimetic nanofibers (8 – 12 nm), and the antibacterial and anti‐inflammatory properties of SAAP148, achieves functional hair follicle regeneration and physiologically aligned collagen I/III ratios. Its triphasic action outperforms clinical benchmarks: sub‐20 s clotting, sustained antimicrobial release, and immune‐evasive regeneration. CRS redefines biomaterial design, offering an off‐the‐shelf, antibiotic‐free solution that meets antimicrobial goals while achieving epithelialization (> 98% closure on day 12). This work addresses major medical challenges in regenerative immunology and antimicrobial resistance with the synergistic action of peptides and proteins, potentially providing new ideas for future protein‐peptide drug combinations.

## 5 Experimental Section

### Construction of the Nano‐Micro Hydrogel Scaffolds

Type I collagen was extracted from rat caudal tendons using an acid extraction method (To obtain a higher concentration and mechanical strength of collagen, the Col I was extracted by ourselves, Figure S15, Supporting Information). This process involved repeated dissolution in a 0.1% acetic acid solution followed by salting out in a 2 mol L^−1^ NaCl solution. The purified collagen was then stored at 4 °C. The hemostatic peptide RADA16 (RADARADARADARADA), an amphiphilic polypeptide composed of a repeating sequence of positively charged arginine (R), negatively charged aspartic acid (D), and hydrophobic alanine (A), was chemically synthesized (HPLC and MS data are provided in Figure S1, Supporting Information). Additionally, the antimicrobial peptide SAAP148 (Ac‐LKRVWKRVFKLLKRYWROLKKPVR‐NH_2_) was screened and chemically synthesized (HPLC and MS data are provided in Figure S2, Supporting Information). A micro‐nano porous fiber substrate material (Col I + RADA16, referred to as CR) was fabricated using rat type I collagen (Col I, 20 mg mL^−1^) as the base material and the hemostatic peptide RADA16 (10 mg mL^−1^), which self‐assembles into a nano‐fibrous structure. This micro‐nano scaffold was subsequently loaded with the antimicrobial peptide SAAP148 (30 µg mL^−1^). Col I self‐assembled with RADA16 and SAAP148 (Col I + RADA16 + SAAP148, referred to as CRS) under weakly acidic conditions (pH 4.0), which prevented collagen and peptide aggregation.

### Structure of the Col I, RADA16, and SAAP148

The geometrical molecular optimization and imaging of RADA16/SAAP148 were performed using Gaussian 09 program.^[^ ^53^ ^]^ The secondary structures of Col I, RADA16, and SAAP148 proteins were analyzed using Fourier transform infrared spectroscopy (FTIR, Thermo Fisher Scientific Nicolet iS20, USA) and circular dichroism (CD) spectroscopy (Chirascan, UK). The protein solutions (0.3 mg mL^−1^) were scanned for CD signals in the wavelength range of 190–250 nm, respectively.

### Molecular Dynamics Simulation

Molecular dynamics simulations were conducted using GROMACS 2024.2 with the AMBER99SB‐ILDN force field and TIP3P water model. The protein was solvated in a cubic water box (1.0 nm buffer), neutralized with Na⁺/Cl^−^ ions, and energy‐minimized until convergence (< 1000 kJ·mol^−1^·nm^−1^). The system underwent sequential equilibration: 100 ps NVT (300 K, V‐rescale thermostat) with protein position restraints, followed by 100 ps NPT (300 K, 1 bar; Parrinello‐Rahman barostat) with gradual restraint release. A 10 ns production simulation was performed under NPT conditions (2 fs time step), using PME for electrostatics (1.0 nm cutoff for short‐range interactions) and LINCS constraints for hydrogen bonds. Trajectories and energy data were saved every 100 and 10 ps, respectively. The quantitative analysis of conformational stability (backbone RMSD) and structural compactness (Radius of gyration, Rg) was visualized through time‐dependent plots generated in Origin software.

### FTIR of the Col I, CR, and CRS Hydrogel Films

To investigate the different functional groups of the molecules, Col I, CR, and CRS were analyzed using Fourier transform infrared (FTIR) spectroscopy. For sample preparation, pre‐gel solutions of Col I, CR, and CRS were individually applied onto glass slides and air‐dried overnight at ambient temperature. The chemical characterization was subsequently performed using an FTIR spectrometer (Nicolet 5700, Thermo Scientific, USA) to identify characteristic molecular vibrations associated with specific functional groups.

### Cryo‐Electron Microscopy

The Col I, CR, and CRS were individually formulated into 10 mg mL^−1^ aqueous solutions. Subsequent structural analysis was conducted using cryogenic scanning electron microscopy (Cryo‐SEM, SU8100, Hitachi, Japan) equipped with a cryo‐transfer system (PP3010T, Quorum Technologies, UK) to examine the supramolecular architectures of the self‐assembled protein complexes under high‐vacuum conditions.

### Water Contact Angle and Surface Energy Measurement of the Hydrogel Films

The wettability characteristics of Col I, CR, and CRS hydrogels were evaluated through static contact angle measurements. For water contact angle analysis, three distinct droplets were deposited on each hydrogel film and quantified using an optical goniometer (OCA15EC, DataPhysics, Germany). Subsequently, the surface interaction properties were further investigated by measuring static diiodomethane contact angles under identical experimental conditions. Each specimen underwent triplicate droplet measurements to ensure data reproducibility, with all values recorded via the optical contact angle measurement system. Finally, the surface free energy of Col I, CR, and CRS hydrogels were calculated using the OWRK model.

### Observation and Mechanical Property of Hydrogel Scaffolds

The formed different hydrogels (Col I, CR, CRS) were lyophilized by a vacuum freeze drier (Heto, LL3000). After coating with gold by sputter coater (SCD 005, Bal‐Tec, USA), the microstructure of hydrogel scaffolds was observed using a scanning electron microscope (SEM, FEI Quanta 450, USA). Then, the elastic modulus of the hydrogel scaffolds was measured by a Bose dynamic mechanical analysis (DMA, ELF3220, USA).

### The Release of SAAP148 from CRS Hydrogels

SAAP148 labelled with Fluorescein isothiocyanate (FITC) was mixed with Col I+RADA16 pre‐gel solution and then formed a Hybrid hydrogel (Col I hydrogel served as a control). The sustained‐release profile of the SAAP148 peptide was quantitatively assessed through a fluorescence quantification method at predetermined intervals. Spectrofluorometric analysis was conducted utilizing a multifunctional microplate reader (ENVISION2105, PerkinElmer, USA) for precise intensity determination. Throughout the experiment, fresh PBS replenishment was performed at each sampling time point to maintain hydrogel hydration, with cumulative release kinetics systematically recorded and graphically represented.

### The Degradation of the Hydrogels

To simulate the in vivo protein degradation process, dried Col I, CR, and CRS materials were incubated with collagenase solutions containing either Type I (100 U mL^−1^) or Type II collagenase (100 U mL^−1^), respectively, and simulated body fluids (SBF) were used as a control group. All samples were incubated at 37 °C for 4 h, followed by SDS‐PAGE analysis. After electrophoresis, the gels were stained with Coomassie Brilliant Blue R250 and subjected to quantitative statistical analysis using Gel‐Pro Analyzer software.

### Cell Adhesion

Cell adhesion was used to evaluate the cytocompatibility of the different hydrogel scaffolds. Fetal bovine serum used in this article was FBS (Umedium, Hefei, China). Briefly, 1 × 10^6^ L929 cells were seeded in each scaffold and cultured for 2, 4 h at 37 °C, respectively, and then they were fixed with 2.5% glutaraldehyde. The morphology of adhered cells on each scaffold were observed by the SEM (FEI Quanta 450, USA). To quantify the adhesion of cells to the surface of the materials, individual cells were manually outlined using ImageJ. Area and perimeter of the adhered cells were quantified for at least 50 cells per substrate. The cell circularity were calculated using Equation (1).^[^ ^54^ ^]^

(1) $$Cell\;circularity=\frac{4\pi *\;Cell\;area}{Cell\;perimeter^{2}}$$

### Cell Migration

The effect of the Col I, CR, or CRS (20 µg mL^−1^) was evaluated on cell migration, L929 cells were seeded into 12‐well plates. When the cells were fused to more than 90%, lines were drawn on the bottom of the wells, different groups of protein solutions were added to the cells. After culturing for 72 h, crystal violet staining was performed, and the cells were observed and photographed under an optical microscope (*n* = 6). Cells cultured in DMEM only acted as a control (*n* = 6). The migration area at 0 h (A0) and 72 h (At) were measured by ImageJ, and the migration area rate was calculated using Equation (2).

(2) $$Cell\;migration\;area\;\left(\%\right)=\frac{0\;h\;scratch\;area-x\;h\;scratch\;area}{0\;h\;scratch\;area.}*100\%$$

### Cell Proliferation

The effects of different hydrogel materials were evaluated on the proliferation of L929 cells. In 96‐well plates, 1 × 10^3^ cells were added to each well, while 1 × 10^4^ cells were seeded in each well of confocal dishes. Solutions of Col I, CR, and CRS (20 µg mL^−1^) prepared in 3% FBS medium were added, with 3% FBS medium alone used as the control. On days 1, 3, and 5, the medium was removed, and 100 µL of CCK‐8 solution (MI00615A, mishubio) was added to each well, followed by an incubation period of 2 h. The absorbance of the liquid was then measured at OD 450 nm using a microplate reader (BioTek, USA). For the confocal dishes, the medium was discarded, and the wells were washed three times with PBS. Fixation was performed with 4% PFA for over 30 min, followed by fluorescence staining.

### Observation of Live/Dead *E. coli* and *S. aureus*

The staining method was according to the protocol of LIVE/DEAD BacLight Viability Kit (BBcellProbeN01/PI double staining, green/red fluorescence, BB‐41266). Briefly, *E. coli* and *S. aureus* were cultured and reached the logarithmic growth phase (OD600 = 0.6 – 0.8), respectively. Then, the bacterial solution and the sterilized CRS composites containing different concentrations of SAAP148 antimicrobial peptide were co‐cultured for 12 h (220 rpm). Finally, the bacteria were washed and collected by centrifugation (5,000 rpm, 10 min), and then stained by the LIVE/DEAD BacLight Viability Kit and observed by laser confocal fluorescence microscope (FV3000 Olympus, Japan).

### Minimum Inhibitory Concentration (MIC)

The quantitative analysis of bacterial inhibition was carried out by multiplicative dilution method to the lowest concentration MIC of antimicrobial peptide that completely inhibited the growth of bacteria. Briefly, overnight *S. aureus* cultures in Luria‐Bertani (LB) reached the logarithmic phase. Based on the optical density (OD) at 600 nm, the bacterial suspension was diluted to the desired inoculum concentration (OD = 0.09 – 0.14). The final antimicrobial peptide SAAP148 concentration was diluted to 30, 25, 20, 15, 10, 5, 2.5, and 1.25 µg mL^−1^. Fifty microliters PBS and 50 µL bacterial fluid were set up as positive control and 50 µL PBS and 50 µL (2×) LB were set up as negative control (*n* = 6). The OD600 value was measured after incubation at 37 °C for 24 h on a shaker, and the inhibition rate of different concentrations of antimicrobial peptides was calculated according to Equation (3) to derive the lowest concentration of antimicrobial peptides to inhibit bacterial growth.

(3) $$Bacterial\;inhibition\;rate\;\left(\%\right)=\frac{positive\;OD-test\;OD}{positive\;OD-negative\;OD}*100\%$$

### Circle of Inhibition Size

The *S. aureus* bacterial solution was diluted 100‐fold with LB liquid medium and set aside. Subsequently, 200 µL of the bacterial suspension (1 × 10^8^ cfu mL^−1^) was plated on LB agar plates. The circular composite material was used to attach the test and control samples onto the Petri dishes, after the bacterial solution was air‐dried. The dishes were then incubated at 37 °C for 20 h to observe the zones of inhibition, which were then measured to determine the antibacterial effect of the material against *S. aureus*.

### Hemolysis Determination

The hemocompatibility assessment was conducted using fresh anticoagulated rabbit blood standardized to an optical density of 0.8 ± 0.3 at 545 nm (0.2 mL in 10 mL distilled water), with experimental groups (*n* = 3) comprising negative controls (10 mL saline), positive controls (10 mL distilled water), and test samples preconditioned in saline. Following 30 min equilibration at 37 ± 1 °C, 0.2 mL blood suspension was introduced to each tube, incubated for 60 min, then centrifuged (750 g, 5 min) for supernatant analysis. Validated by control absorbance thresholds (negative < 0.03, positive 0.8 ± 0.3). The hemolysis ratio was calculated via Equation (4), with > 5% indicating clinically significant erythrocyte damage potential.

(4) $$Z=\frac{Dt-Dnc}{Dpc-Dnc}\times 100\%$$

In the formula: *Z* ‐ hemolysis ratio;

Dt ‐ the value of the absorbance of hemolysis obtained by the specimen;

Dnc — negative control absorbance value;

Dpc — positive control absorbance value.

### Liver Hemostasis Test

Twenty‐four KM mice (male, 6–8 weeks, 22 – 25 g) were purchased from Experimental Animal Center of Medical College of Xi'an Jiaotong University, and all procedures were approved by the Northwest University IACUC (ACUC2013015). Mice were randomly grouped into control, Col I, CR, and CRS composite groups (*n* = 6/each group). The mice in each group were injected intraperitoneally with 3% sodium pentobarbital (40 mg kg^−1^), anesthetized, fixed, sterilized, and the skin was cut along the midline of the abdomen, and the abdominal cavity was entered layer by layer to expose the liver fully. The surrounding peritoneal fluid was aspirated with sterile gauze, made an incision of 5 mm in length at the distal end of the lobus hepatis sinister. A sample was utilized to cover the incision, and the amount of bleeding was calculated from the difference in weight before and after covering the sample. The bleeding was covered and wiped with sterile gauze as the control group. Meanwhile, the bleeding from the incision was recorded until the bleeding stopped and the hemostasis time was recorded. The samples were fixed with 2.5% glutaraldehyde, dehydrated, dried, coated with gold by a sputter coater (SCD 005, Bal‐Tec, USA), and observed by SEM (FEI Quanta 450, USA).

### Repair of Skin Defects in *S. aureus* Infection

Thirty‐two KM Mice (male, 6 – 8 weeks, 22 – 25 g) were randomly divided into control, Col I, CR, and CRS composite groups (20 mg mL^−1^ Col I, 10 mg mL^−1^ RADA16, 30 µg mL^−1^ SAAP148, *n* = 8/each group). Sterilization and disinfection were required before the composites were applied to the animals. After each group of mice was anesthetized with 3% sodium pentobarbital, the dorsal surface was shaved with a razor. Then, the epidermis and dermis were removed with a perforator to form a 10 mm circular skin wound, and the wound surface was infected with 10 µL of *S. aureus* solution (1 × 10^8^ CFU mL^−1^) to establish an infected wound.^[^ ^55^ ^]^ Then the composites of each group were carefully transferred to the wound. Additionally, pre‐operatively the mice were injected with Carprofen and during the operation, the mice were anesthetized with 2.5% isoflurane in oxygen. A heated pad was then used to maintain the body temperature after the operation. The area of the wound was recorded every day. On day 1, 3, 5, 7, and 9, the wound surface was smeared with a moistened sterile cotton swab and incubated with 1 mL of LB medium at 37 °C for 6 h. The cultured bacterial solution was diluted 10^3^‐fold, and 200 µL of the bacterial solution was spread on LB agar medium. After incubation at 37 °C for 10 – 12 h, the wound was photographed and recorded. Mice were euthanized on days 3, 7, 12, and 18, respectively.

### Histological Staining and Spatial Proteomics Analysis of Infected Wound Healing Process

To observe skin regeneration during wound repair, hematoxylin eosin (HE), Masson's trichrome staining (MTC), Elastic von gieson staining (EVG) and Picro sirius red staining (PSR) were used for histopathological analysis to evaluate the wound healing process. Meanwhile, the distribution of Vimentin (host: mouse, proteintech, 60330‐1‐Ig), type I collagen (host: rabbit, Abcam, AB138492), CK14 (host: rabbit, abclonal, A25205), CK5 (host: rabbit, abclonal, A11396), CD31 (host: rabbit, proteintech, 28083‐1‐AP), α‐SMA (host: mouse, proteintech, 67735‐1‐Ig), PGP9.5 (host: rabbit, abclonal, A19101), CD86 (host: rabbit, proteintech, 26903‐1‐AP), CD163 (host: rabbit, proteintech, 16646‐1‐AP), CD44 (host: rabbit, abclonal, A19020), Lamin B1 (host: mouse, proteintech, 66095‐1‐Ig), CoraLite488‐conjugated Goat Anti‐Rabbit IgG(H+L) (proteintech, SA00013‐2) and CoraLite594‐conjugated Goat Anti‐Mouse IgG(H+L) (proteintech, SA00013‐3) in the regenerated skin was observed by immunofluorescence staining. To quantify the content of various growth factors and inflammatory factors in the regenerated skin, the frozen samples taken were subjected to RT‐qPCR (Primers in Table S1, Supporting Information). Intergroup differences in the skin healing process were analyzed by spatial proteomics. The main steps were to select the healing skin tissue regions in the samples after H&E staining, cut the selected regions for mass spectrometry, and search the library for analysis after the detection according to the previous study.^[^ ^56^ ^]^

### Analysis of the Effect of CRS Hydrogels on Macrophage Polarization

Raw264.7 cells were cultured and seeded in 6‐well plates at a density of 5 × 10^5^ cells well^−1^. To induce macrophage polarization from M0 to M1 phenotype, cells were treated with 100 ng mL^−1^ LPS.^[^ ^57^, ^58^ ^]^ The experimental design included: i) a blank group (untreated control), ii) an LPS‐only positive control group, and iii) three treatment groups receiving Col I, CR, or CRS solutions (200 µg mL^−1^ Col I, 100 µg mL^−1^ RADA16, 30 ng mL^−1^ SAAP148), respectively. Following 6‐h incubation at 37 °C, the culture medium was aspirated and cells were washed thrice with PBS. First, the M0 and M1‐polarized macrophages were observed after the treatment through immunofluorescence (Labeled by CL488‐Phalloidin, 1:100, proteintech, PF00001). Then total RNA and protein were subsequently extracted using Trizol reagent and RIPA lysis buffer for RT‐qPCR (Primers in Table S1, Supporting Information) and Western blotting (WB), respectively.

WB analysis was performed to evaluate iNOS expression in M1‐polarized macrophages across control and experimental groups, with GAPDH as an internal control. Briefly, membrane‐bound proteins were probed with primary antibodies (anti‐iNOS, 1:2000, abclonal, A3774; anti‐GAPDH, 1:50 000, proteintech, 60004‐1‐Ig), followed by incubation with HRP‐conjugated secondary antibodies (anti‐rabbit, 1:5000, abclonal, AS014; donkey anti‐mouse, 1:20 000, abclonal, AS033). Protein bands were subsequently visualized using enhanced chemiluminescence (ECL) reagents and imaged with a chemiluminescence imaging system (ChemiScope 6200, Clinx, China).

### Statistical Analysis

The data were analyzed using GraphPad Prism 10.0 software. Pairwise comparisons between groups were performed using an unpaired student's T‐test or one‐way analysis of variance (ANOVA) with post‐hoc Tukey HSD test. Statistically significant differences were set at *p < 0.05.

## Conflict of Interest

The authors declare no conflict of interest.

## Author Contributions

Q.L. and J.W. contributed equally to this work. Q.L. and J.W.W. designed and performed the study, collected and analyzed data, and prepared the manuscript. H.B. analyzed data and prepared the manuscript. Y.F.C. performed animal experiments. Y.F.Z. and X.L.P. performed cell assays and guided histological analysis. F.L.C. supervised the project. X.Z. prepared and revised the manuscript. Z.Y.C. supervised the project, prepared and revised the manuscript.

## Supporting information



Supporting Information

## Data Availability

Research data are not shared.

## References

[1] K. Kabashima , T. Honda , F. Ginhoux , G. Egawa , Nat. Rev. Immunol. 2019, 19, 19. 30429578 10.1038/s41577-018-0084-5

[2] C. Zhang , G. R. Merana , T. Harris‐Tryon , T. C. Scharschmidt , Mucosal Immunol. 2022, 15, 551. 35361906 10.1038/s41385-022-00505-y

[3] A. Szlauer‐Stefańska , G. Kamińska‐Winciorek , S. Giebel , M. Bagłaj , Adv. Clin. Exp. Med. 2020, 29, 1221. 33064381 10.17219/acem/126739

[4] R. Kalsi , F. Messner , G. Brandacher , Curr. Opin. Organ Transplant. 2020, 25, 464. 32773504 10.1097/MOT.0000000000000798

[5] K. S. Vyas , H. C. Vasconez , Healthcare (Basel) 2014, 2, 356. 27429283 10.3390/healthcare2030356 PMC4934597

[6] K. Cheng , Y. Deng , L. Qiu , S. Song , L. Chen , L. Wang , Q. Yu , Smart Mater. Med. 2024, 5, 240.

[7] X. Chen , X. Li , W. He , M. Wang , A. Gao , L. Tong , S. Guo , H. Wang , G. Pan , Innovation 2023, 4, 100483. 37560332 10.1016/j.xinn.2023.100483 PMC10407542

[8] H. Li , X. Lin , S. Rao , G. Zhou , L. Meng , Y. Yu , J. Wang , X. Chen , W. Sun , Research 2024, 7, 0445. 39109247 10.34133/research.0445 PMC11301524

[9] J.‐C. Lv , X. Yang , Z.‐L. Zheng , Z.‐G. Wang , R. Hong , Y. Liu , E. Luo , J.‐X. Gou , L. Li , B. Yuan , ACS Appl. Mater. Interfaces 2024, 17, 776. 39689966 10.1021/acsami.4c20219

[10] Y. Wang , K. Liu , W. Wei , H. Dai , Adv. Funct. Mater. 2024, 34, 2402531.

[11] W. Zhang , X. Shen , W. Li , J. Wang , Z. Kuang , L. Gui , Y. Tao , P. Song , F. Ge , L. Zhu , ACS Appl. Nano Mater. 2023, 6, 20556.

[12] Y. Xiong , L. Chen , P. Liu , T. Yu , C. Lin , C. Yan , Y. Hu , W. Zhou , Y. Sun , A. C. Panayi , Small 2022, 18, 2104229. 10.1002/smll.202104229 34791802

[13] M. Salamito , V. Haydont , H. Pageon , F. Ruggiero , S. Girardeau‐Hubert , Matrix Biol. 2025, 140, 133. 40716529 10.1016/j.matbio.2025.07.006

[14] Y. Guo , N. Cheng , H. Sun , J. Hou , Y. Zhang , D. Wang , W. Zhang , Z. Chen , Front. Bioeng. Biotechnol. 2023, 10, 1062676. 36714615 10.3389/fbioe.2022.1062676 PMC9873964

[15] F. Gelain , Z. Luo , M. Rioult , S. Zhang , NPJ Regener. Med. 2021, 6, 9. 10.1038/s41536-020-00116-w PMC7889856 33597509

[16] M. Naghavi , S. E. Vollset , K. S. Ikuta , L. R. Swetschinski , A. P. Gray , E. E. Wool , G. R. Aguilar , T. Mestrovic , G. Smith , C. Han , Lancet 2024, 404, 1199.39299261

[17] K. E. Ridyard , J. Overhage , Antibiotics 2021, 10, 650. 34072318 10.3390/antibiotics10060650 PMC8227053

[18] A. de Breij , M. Riool , R. A. Cordfunke , N. Malanovic , L. de Boer , R. I. Koning , E. Ravensbergen , M. Franken , T. van der Heijde , B. K. Boekema , Sci. Transl. Med. 2018, 10, aan4044. 10.1126/scitranslmed.aan4044 29321257

[19] M. Adélaïde , E. Salnikov , F. Ramos‐Martín , C. Aisenbrey , C. Sarazin , B. Bechinger , N. D'amelio , Pharmaceutics 2023, 15, 761. 36986623 10.3390/pharmaceutics15030761 PMC10051583

[20] Y. Luo , Y. Song , Int. J. Mol. Sci. 2021, 22, 11401.34768832

[21] L. Gan , Y. Chi , Y. Peng , S. Li , H. Gao , X. Zhang , S. Ji , Z. Feng , S. Zhang , Int. J. Mol. Sci. 2024, 25, 11776. 39519326 10.3390/ijms252111776 PMC11546786

[22] N. Mookherjee , K. L. Brown , D. M. E. Bowdish , S. Doria , R. Falsafi , K. Hokamp , F. M. Roche , R. Mu , G. H. Doho , J. Pistolic , J. Immunol. 2006, 176, 2455. 16456005 10.4049/jimmunol.176.4.2455

[23] R. M. Sarate , J. Hochstetter , M. Valet , A. Hallou , Y. Song , N. Bansaccal , M. Ligare , M. Aragona , D. Engelman , A. Bauduin , Cell 2024, 187, 5298. 39168124 10.1016/j.cell.2024.07.031

[24] W. Nie , X. Yuan , J. Zhao , Y. Zhou , H. Bao , Carbohydr. Polym. 2013, 96, 342. 23688490 10.1016/j.carbpol.2013.04.008

[25] Z. Chen , R. Wang , J. He , Q. Liu , Y. Zhang , Y. Wang , L. Liu , M. Song , F. Chen , ACS Appl. Bio Mater. 2024, 8, 236. 10.1021/acsabm.4c01096 39723909

[26] B. Guo , R. Dong , Y. Liang , M. Li , Nat. Rev. Chem. 2021, 5, 773. 37117664 10.1038/s41570-021-00323-z

[27] V. Panwar , A. Sharma , J. Thomas , V. Chopra , S. Kaushik , A. Kumar , D. Ghosh , Materialia 2019, 7, 100373.

[28] S. Sankar , K. O'Neill , M. Bagot D'Arc , F. Rebeca , M. Buffier , E. Aleksi , M. Fan , N. Matsuda , E. S. Gil , L. Spirio , Front. Bioeng. Biotechnol. 2021, 9, 679525. 34164387 10.3389/fbioe.2021.679525 PMC8216384

[29] L. D. Lozeau , J. Grosha , D. Kole , F. Prifti , T. Dominko , T. A. Camesano , M. W. Rolle , Acta Biomater. 2017, 52, 9. 28017866 10.1016/j.actbio.2016.12.047

[30] A. Albayrak , D. M. Fakioğlu , E. Şenol , Eur. J. Hospital Pharm. 2020, 27, 121. 10.1136/ejhpharm-2019-001873 PMC7043256 32133141

[31] M. E. van Gent , T. van Baaren , S. N. Kłodzińska , M. Ali , N. Dolezal , B. R. van Doodewaerd , E. Bos , A. M. de Waal , R. I. Koning , J. W. Drijfhout , Pharmaceutics 2023, 15, 429. 36839751 10.3390/pharmaceutics15020429 PMC9967827

[32] N. Arora , K. Thangavelu , G. N. Karanikolos , Front. Chem. 2020, 8, 412. 32671014 10.3389/fchem.2020.00412 PMC7326054

[33] Z. Chen , Y. Huang , H. Xing , T. Tseng , H. Edelman , R. Perry , T. R. Kyriakides , Matrix Biol. 2024, 127, 38. 38325441 10.1016/j.matbio.2024.02.001 PMC10958762

[34] S. Ding , S. He , K. Ye , X. Shao , Q. Yang , G. Yang , Int. J. Biol. Macromol. 2023, 253, 127151. 37778580 10.1016/j.ijbiomac.2023.127151

[35] M. Costa , R. P. Pirraco , M. T. Cerqueira , R. L. Reis , A. P. Marques , Stem Cell Heterogeneity, Springer, New York, 2016, pp. 219–226.

[36] D. A. Taylor , L. C. Sampaio , Z. Ferdous , A. S. Gobin , L. J. Taite , Acta Biomater. 2018, 74, 74. 29702289 10.1016/j.actbio.2018.04.044

[37] X. Zhang , X. Chen , H. Hong , R. Hu , J. Liu , C. Liu , Bioact. Mater. 2022, 10, 15. 34901526 10.1016/j.bioactmat.2021.09.014 PMC8637010

[38] Q. Sun , F. Pei , M. Zhang , B. Zhang , Y. Jin , Z. Zhao , Q. Wei , Adv. Sci. 2023, 10, 2204479. 10.1002/advs.202204479 PMC9875655 36382560

[39] T. I. Zarembinski , A. Skardal , T. I. Zarembinski , A. Skardal , HyStem: A Unique Clinical Grade Hydrogel for Present and Future Medical Applications, 5th ed. IntechOpen, London, 2018.

[40] J.‐Y. Xiang , L. Kang , Z.‐M. Li , S.‐L. Tseng , L.‐Q. Wang , T.‐H. Li , Z.‐J. Li , J.‐Z. Huang , N.‐Z. Yu , X. Long , World J. Stem Cells 2024, 16, 334. 38690516 10.4252/wjsc.v16.i4.334 PMC11056631

[41] F. Azimi‐Bahnamiri , H. Mokhtari , S. Khalilollah , S. V. Soltanahmadi , M. Omraninava , R. A. Disfani , M. S. Mirzaie , H. Ranjbaran , R. Javan , M. Shooraj , J. Tissue Viability 2024, 33, 18. 38042701 10.1016/j.jtv.2023.11.008

[42] M. E. A. B. Corrêa , C. Mendes , J. V. S. Bittencourt , A. Takejima , I. C. de Souza , S. C. D. de Carvalho , I. G. Orlandini , T. A. M. de Andrade , L. C. Guarita‐Souza , P. C. L. Silveira , Ann. Biomed. Eng. 2022, 50, 1895. 35802205 10.1007/s10439-022-03008-w

[43] T. Davenport , S. Stavrides , A. Agrawal , M. R. Borrelli , Plast. Reconstruct. Surg.–Global Open 2024, 12, 6369. 10.1097/GOX.0000000000006369 PMC11661756 39712373

[44] H. Haidari , S. Garg , K. Vasilev , Z. Kopecki , A. J. Cowin , Wound Pract. Res.: J. Aust. Wound Manage. Assoc. 2020, 28, 173.

[45] X. Li , X. Chen , L. Guan , W. He , W. Yin , D. Ye , J. Gao , M. Wang , G. Pan , ACS Appl. Mater. Interfaces 2024, 16, 32104. 38865210 10.1021/acsami.4c05967

[46] W. Li , N. Chi , R. A. C. Rathnayake , R. Wang , Biochem. Biophys. Res. Commun. 2021, 560, 66. 33975247 10.1016/j.bbrc.2021.04.088 PMC8165026

[47] S. Lefkopoulos , Nat. Cell Biol. 2023, 25, 1561. 10.1038/s41556-023-01292-9 37945824

[48] Z. Li , K. Lin , Y. Wang , J. Mao , Y. Yin , Z. Li , F. Wang , X. Zeng , Q. Li , X. Wang , Int. Immunopharmacol. 2025, 151, 114352. 40022821 10.1016/j.intimp.2025.114352

[49] L. Lei , G. Wan , X. Geng , J. Sun , Y. Zhang , J. Wang , C. Yang , Z. Pan , J. Ethnopharmacol. 2023, 307, 116193. 36746295 10.1016/j.jep.2023.116193

[50] L.‐J. Zhang , Keratins in Skin Epidermal Development and Diseases, IntechOpen, Rijeka, Croatia, 2018.

[51] Y. Guo , C. J. Redmond , K. A. Leacock , M. V. Brovkina , S. Ji , V. Jaskula‐Ranga , P. A. Coulombe , Elife 2020, 9, 53165. 10.7554/eLife.53165 PMC7250575 32369015

[52] K. Zhou , C. Wu , W. Cheng , B. Zhang , R. Wei , D. Cheng , Y. Li , Y. Cao , W. Zhang , Z. Yao , Cell Death Dis. 2024, 15, 252. 38589352 10.1038/s41419-024-06626-5 PMC11001918

[53] J. Zhang , T. Lu , Phys. Chem. Chem. Phys. 2021, 23, 20323. 34486612 10.1039/d1cp02805g

[54] Z. Chen , J. Zhang , F. Y. Lee , T. R. Kyriakides , Acta Biomater. 2024, 186, 85. 39134130 10.1016/j.actbio.2024.08.011 PMC11500023

[55] C. Zhou , C. Sheng , J. Chen , Y. Liang , Q. Liu , P. Li , X. Huang , B. Liu , Chem. Eng. J. 2022, 450, 138200.

[56] J. Li , J. Ma , Q. Zhang , H. Gong , D. Gao , Y. Wang , B. Li , X. Li , H. Zheng , Z. Wu , Nat. Commun. 2022, 13, 4012. 35817779 10.1038/s41467-022-31659-9 PMC9273758

[57] Y. Ma , X. Liu , R. Dai , Q. Li , C. Y. Cao , Stem Cell Res. Ther. 2024, 15, 469. 39696668 10.1186/s13287-024-04075-7 PMC11656940

[58] H. Zhuo , X. Zhang , M. Li , Q. Zhang , Y. Wang , Antibiotics 2022, 11, 754. 35740160 10.3390/antibiotics11060754 PMC9220503

