# Supplementary material for: RADA16 and SAAP148 Peptide‐Modified Collagen Self‐Assembled Hydrogels for Accelerated Healing of Infected Wounds
Source: Adv Sci (Weinh). 2025 Nov 16;13(6):e19504. doi: 10.1002/advs.202519504 (PMC12866832; doi:10.1002/advs.202519504)
Supplement: Supplementary file 1 — Supporting Information [file ADVS-13-e19504-s001.docx]

Supplementary Information

**RADA16 and SAAP148 Peptide-modified Collagen Self-assembled Hydrogels for Accelerated Healing of Infected Wounds**

Qian Liu ^a 1^, Jiawei Wu ^a 1^, Ho-Pan Bei ^b^, Yufei Chen ^a^, Yifan Zhang ^a^, Xueliang Peng ^a^, Fulin Chen ^a^, Xin Zhao ^b^ *, Zhuoyue Chen ^a^ *

^a^ *Key Laboratory of Resource Biology and Biotechnology in Western China, Ministry of Education, Provincial Key Laboratory of Biotechnology, College of Life Sciences, Northwest University, 229 North Taibai Road, Xi’an, Shaanxi Province 710069, China*

^b^ *Department of Applied Biology and Chemical Technology, The Hong Kong Polytechnic University, 11 Yuk Choi Rd, Hung Hom, Hong Kong SAR 999077, China*

*^c^ Research Institute for Intelligent Wearable Systems, The Hong Kong Polytechnic University, Hung Hom, Kowloon, Hong Kong SAR 999077, China.*

⁎ Corresponding author.

E-mail address: xin.zhao@polyu.edu.hk (X. Zhao), zychen@nwu.edu.cn (Z. Chen).

1 These authors contributed equally to this work.


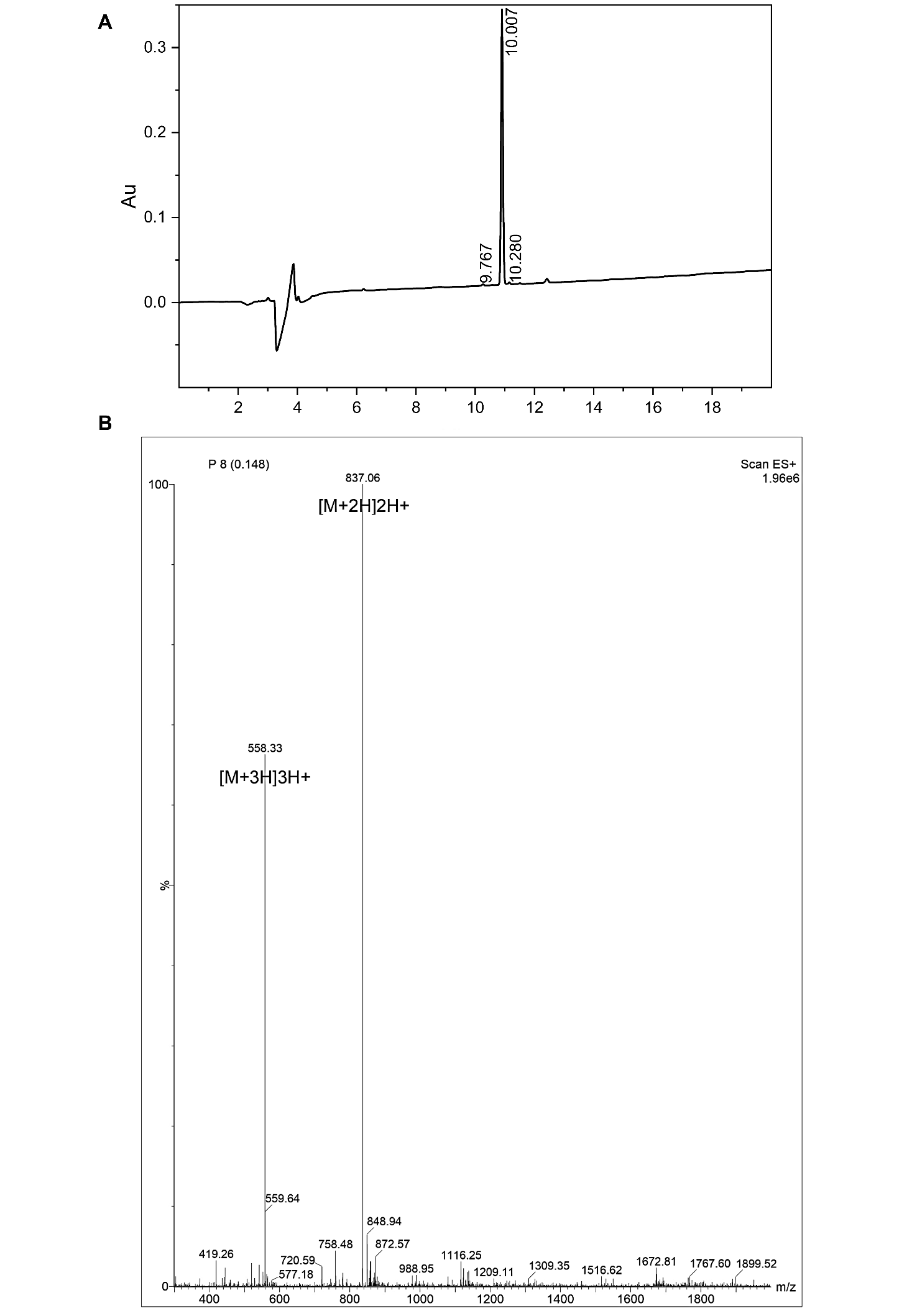


**Figure S1.** A) High performance liquid chromatography (HPLC), and B) mass spectrometry (MS) results of RADA16.


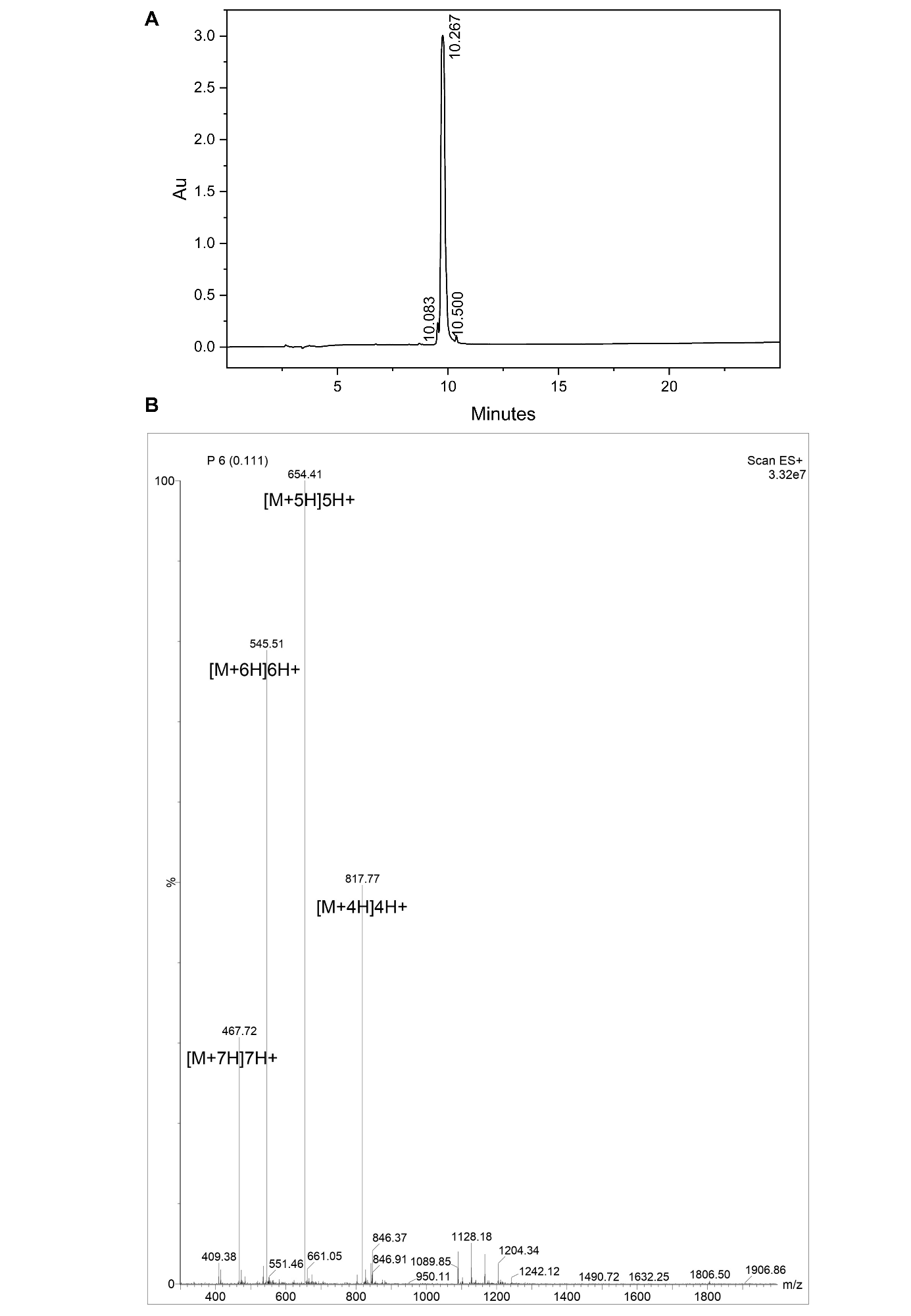


**Figure S2.** A) HPLC and B) MS spectra of SAAP148.

**
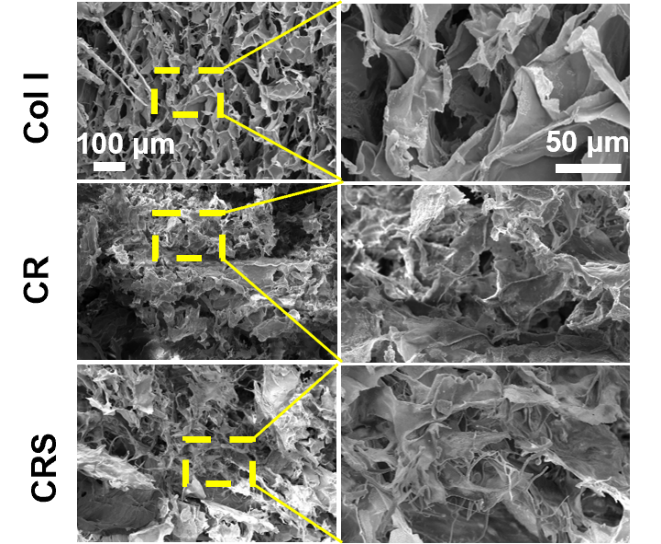
**

**Figure S3.** SEM observation of purified Col I, CR, and CRS hydrogel scaffolds.

**
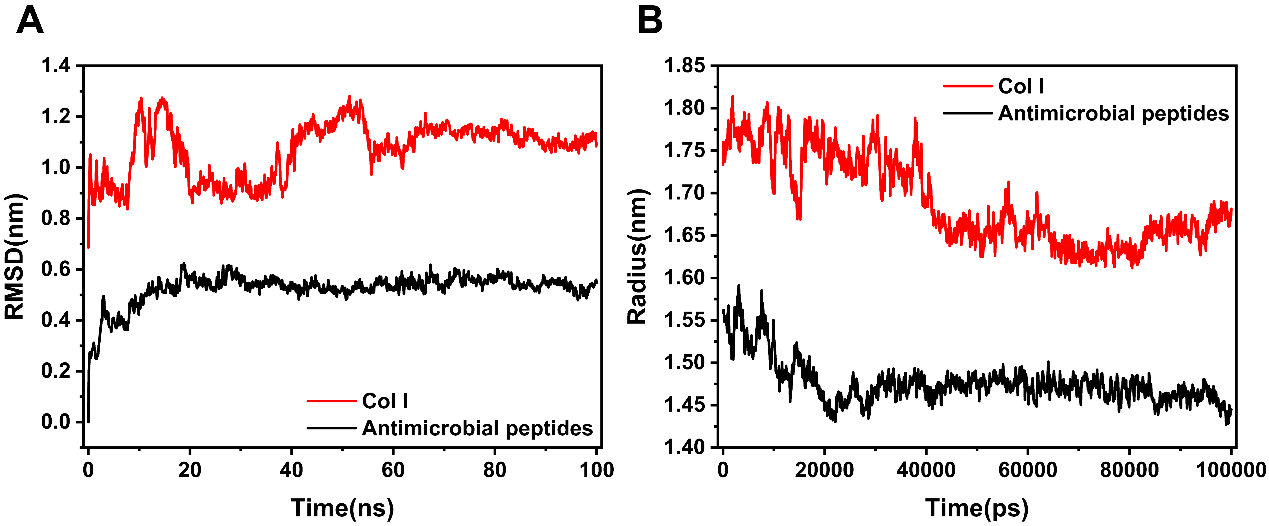
**

**Figure S4.** Molecular dynamic simulation of Col I and antimicrobial peptides. A) Root Mean Square Deviation (RMSD) values. B) Radius of Gyration (Rg) values.


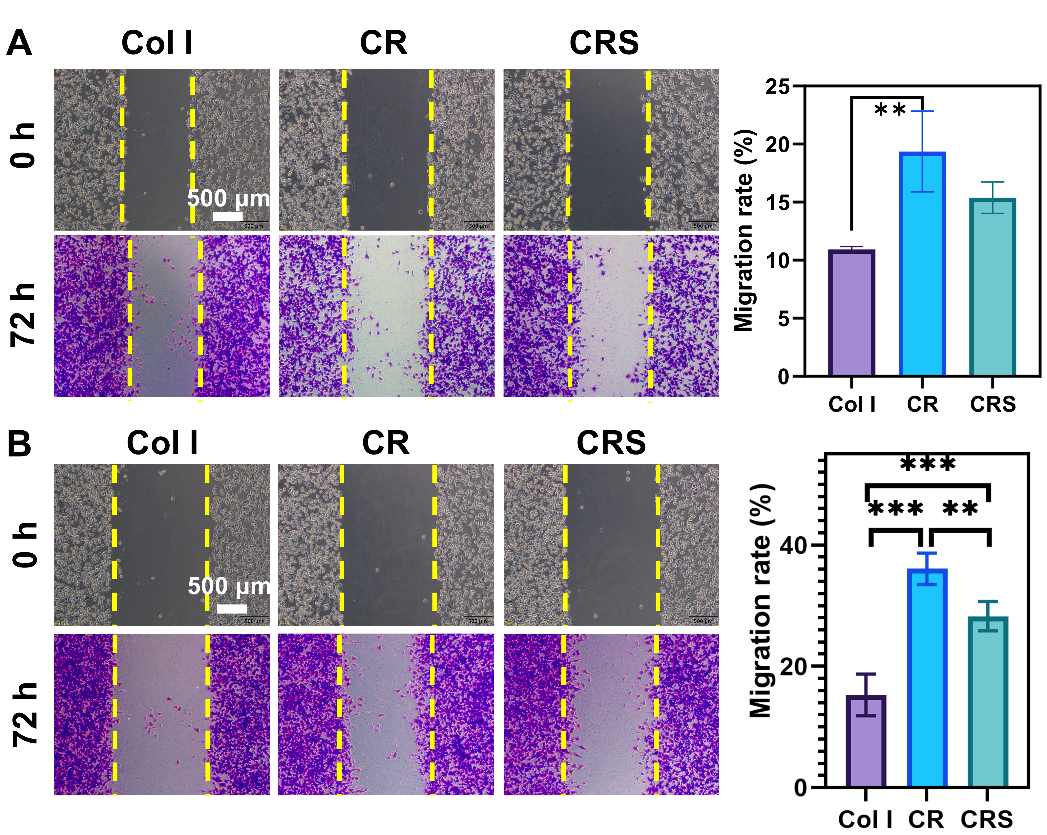


**Figure S5.** Cell Migration assay. L929 migration when co-cultured with the Col I, CR and CRS at A) 10 μg/mL, B) 20 μg/mL for 3 days. Migration rate was evaluated by crystal violet staining and Image J. Mean ± SD, **p < 0.01, ***p < 0.001, n = 3.


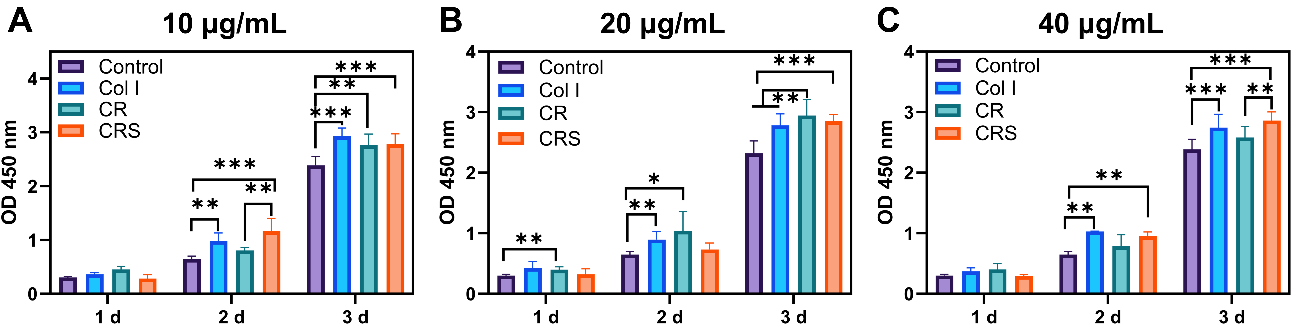


**Figure S6.** Cell proliferation assay. L929 cells (2 × 10^3^ cells/well) were co-cultured with A) 10 μg/mL, B) 20 μg/mL, and C) 40 μg/mL protein solutions (concentration refers to Col I, Col I: RADA16: SAAP148 = 2000: 1000: 3) for 1, 2, and 3 days, respectively, and analyzed by CCK8 Kit. Mean ± SD, *p < 0.05, ** p < 0.01, ***p < 0.001, n = 3.


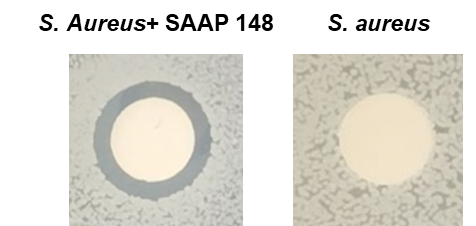


**Figure S7.** SAAP-148 is added to a plate coated with *Staphylococcus aureus* for culture, and the resulting inhibition zone.


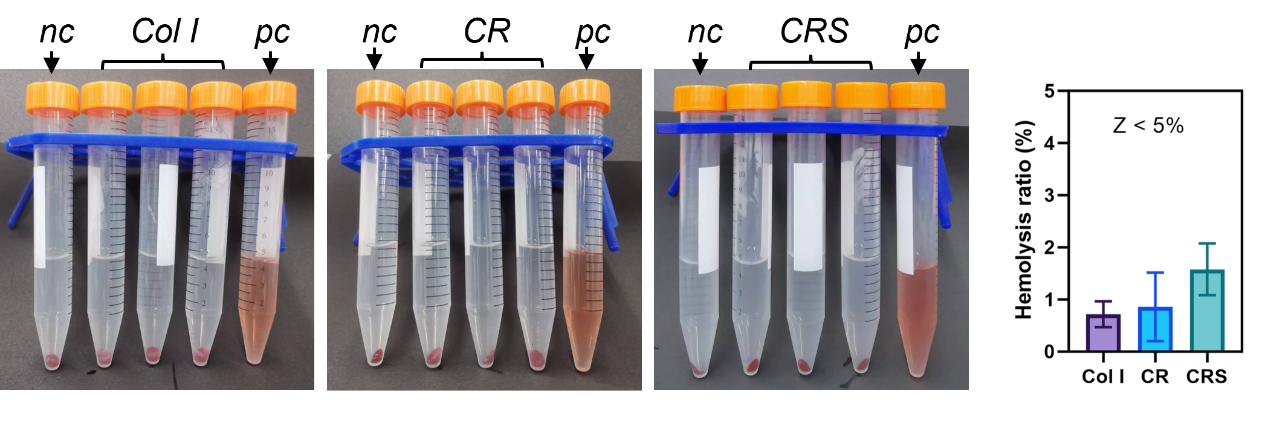


**Figure S8.** Hemolysis evaluation and statistical analysis of the hemolysis rate were performed on Col I, CR, and CRS scaffolds.

**
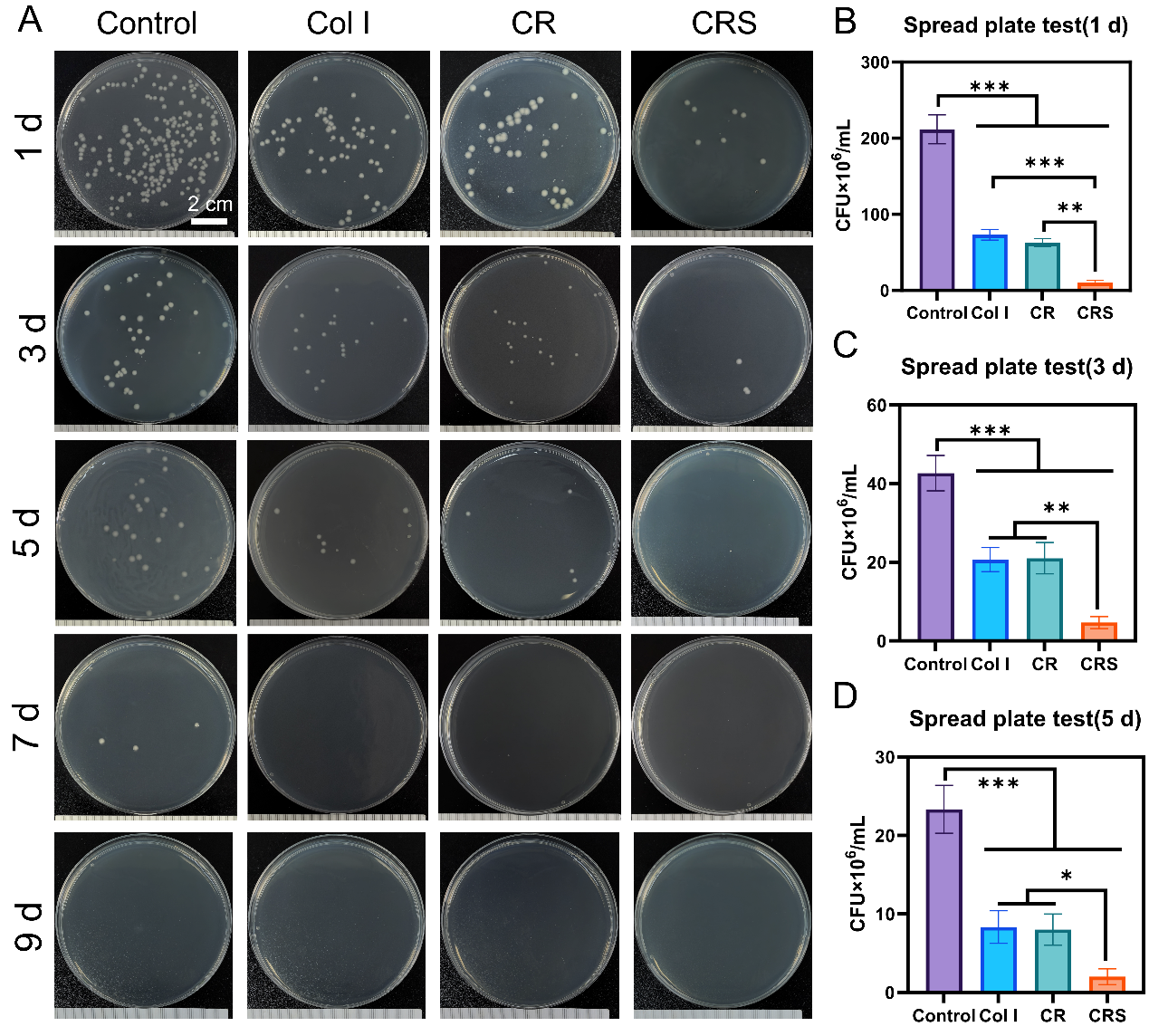
**

**Figure S9.** *In vivo* antibacterial efficacy of the CRS scaffold. A) Bacterial colony formation on LB agar (1 d, 3 d, 5 d, 7 d, and 9 d); B-D) Colony-forming unit (CFU) counts using standard plate counts (1 d, 3 d, and 5 d). Mean ± SD, *p < 0.05, **p < 0.01, ***p < 0.001, n = 3.


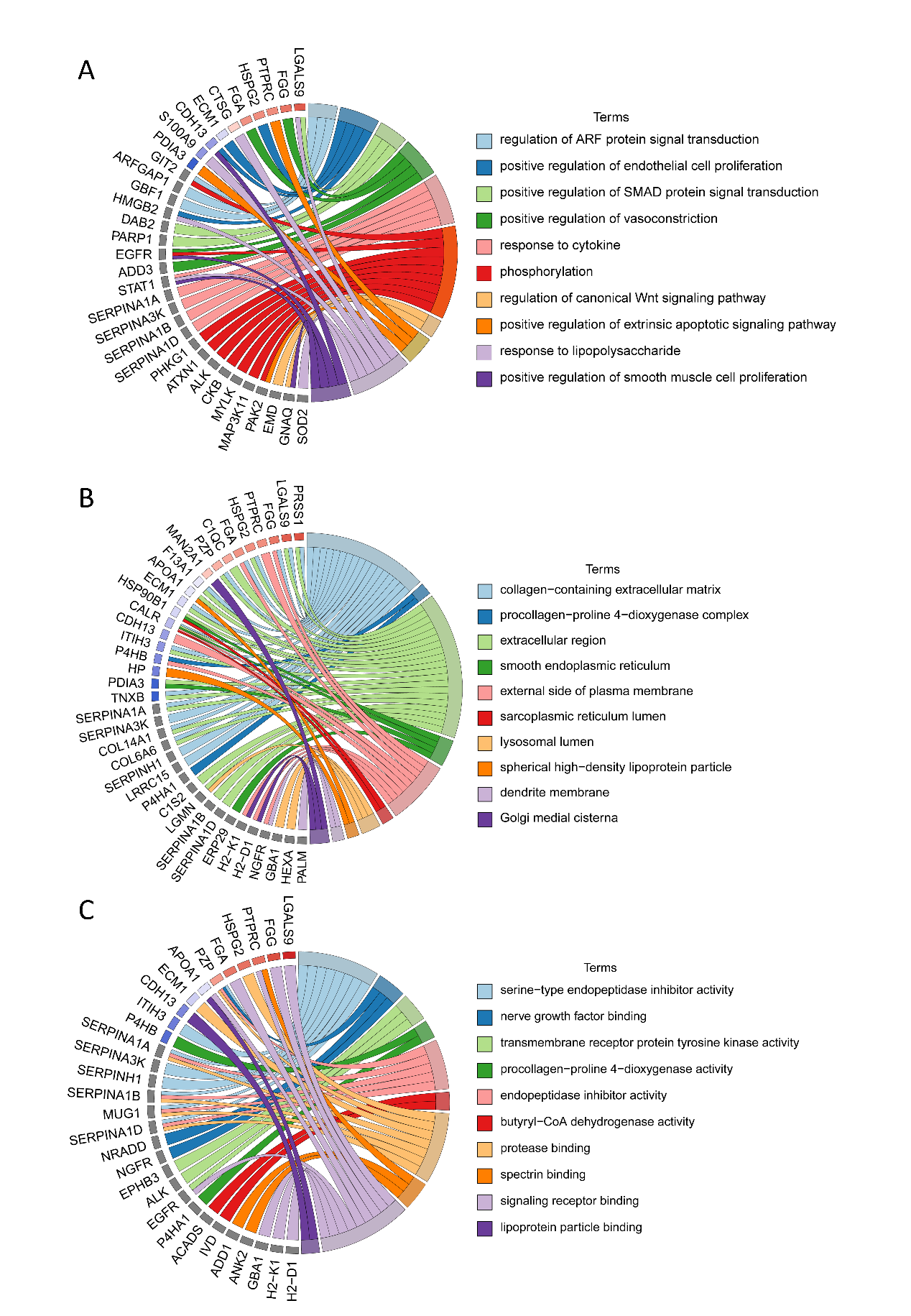


**Figure S10.** GO enrichment analysis chord diagram. The GO analysis results of all differential proteins in 18-day control and CRS tissue samples are shown as A) Biological Process, B) Cellular Component, and C) Molecular Function.

**Figure S11.** Statistical analysis of the fluorescence intensity of CK5 in control, Col I, CR, and CRS groups on post-operative days 12 and 18. Mean ± SD, ***p < 0.001, n = 3.


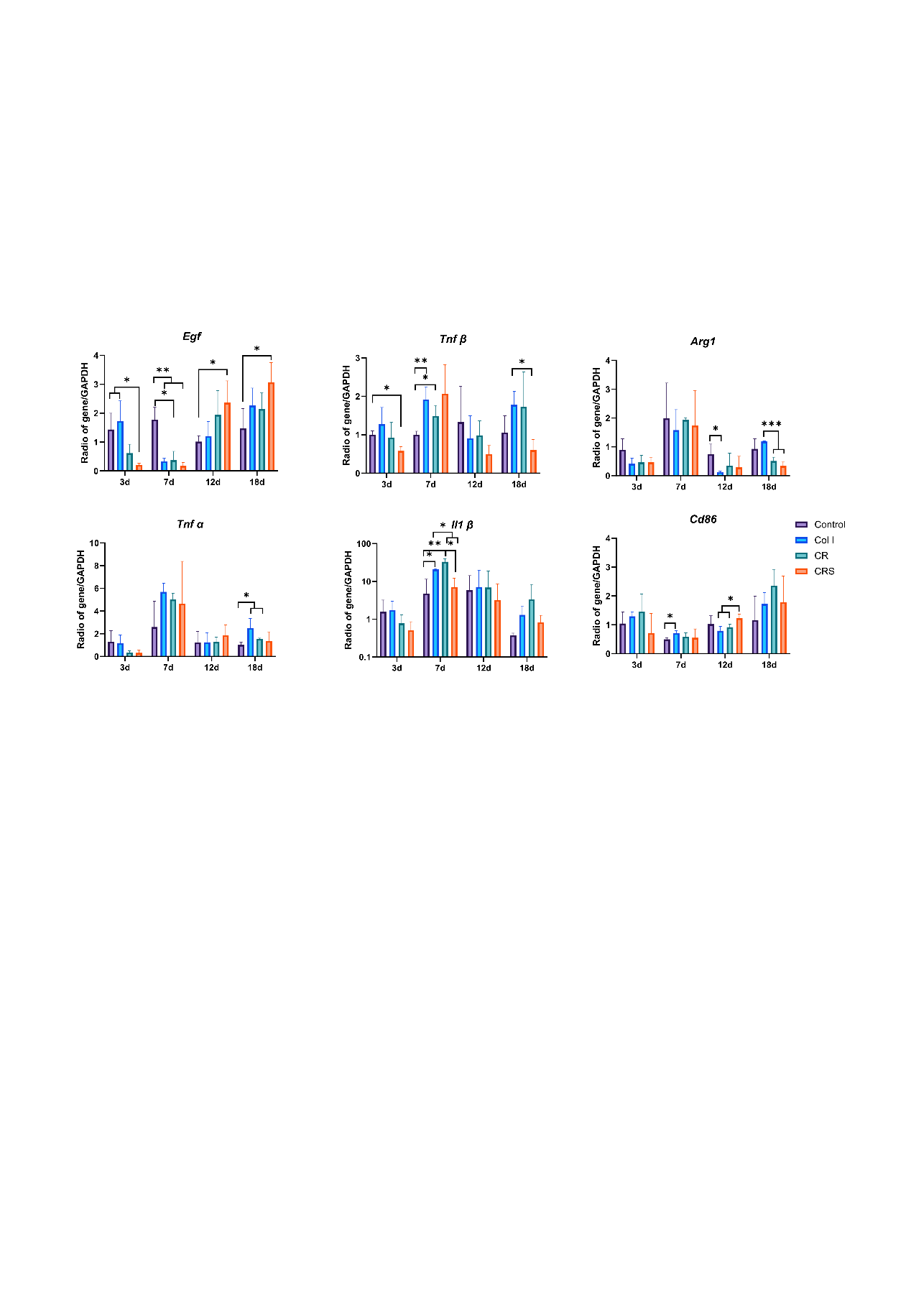


**Figure S12.** The expression of gene levels of *Egf, Tnf β, Tnf α, Arg1, Il 1β*, and *Cd86* at various time points in skin damage repair in COL I, CR, and CRS scaffold groups was measured by qPCR experiments. Mean ± SD, *p < 0.05, **p < 0.01, ***p < 0.001, n = 3.

**
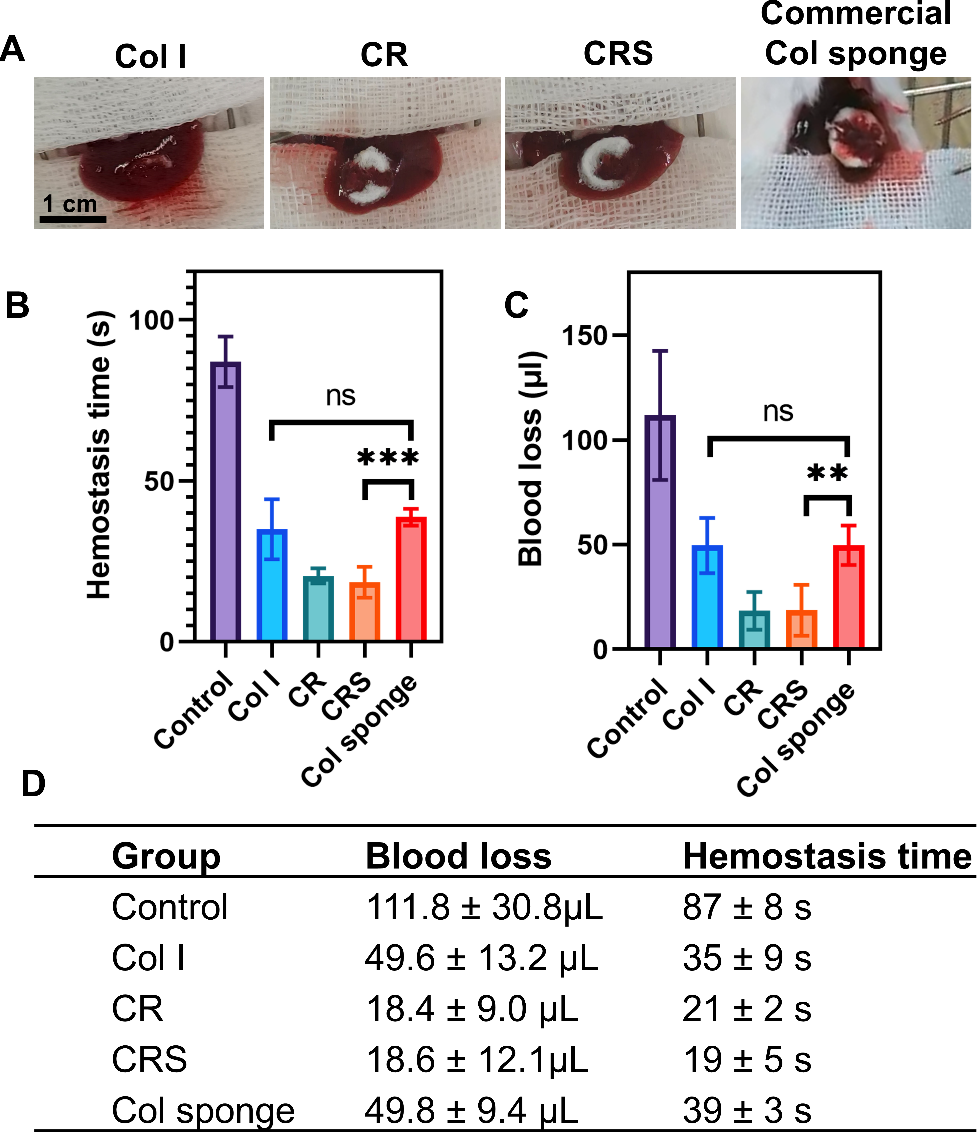
**

**Figure S13.** *In vivo* hemostatic performances of Col I, CR, CRS and commercial Col sponge. A) Optical photographs, B) The amount of hepatic hemorrhage and C) bleeding time, (n = 4, ^ns^p > 0.05, **p < 0.01, ***p < 0.001). D) Average ± SD of blood loss and hemostasis time in each group.


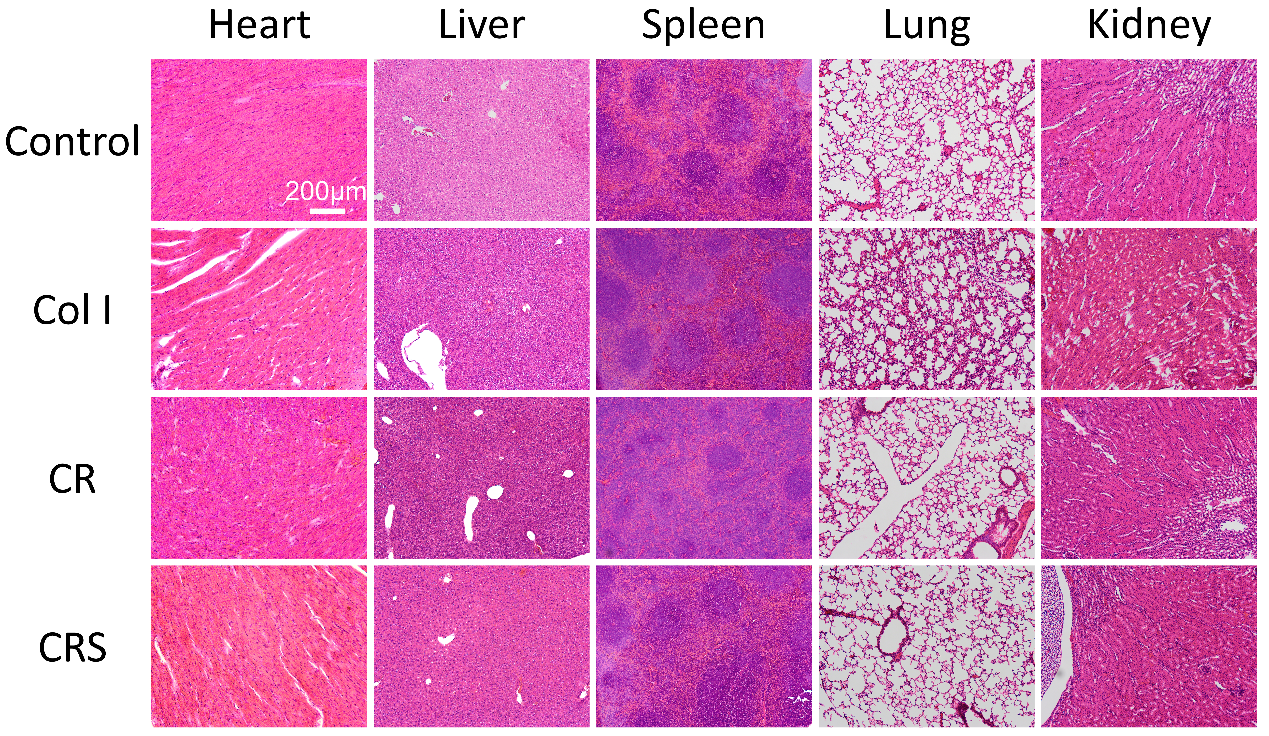


**Figure S14.** HE staining of the heart, liver, spleen, lung, and kidney of mice after implantation of the materials.

**
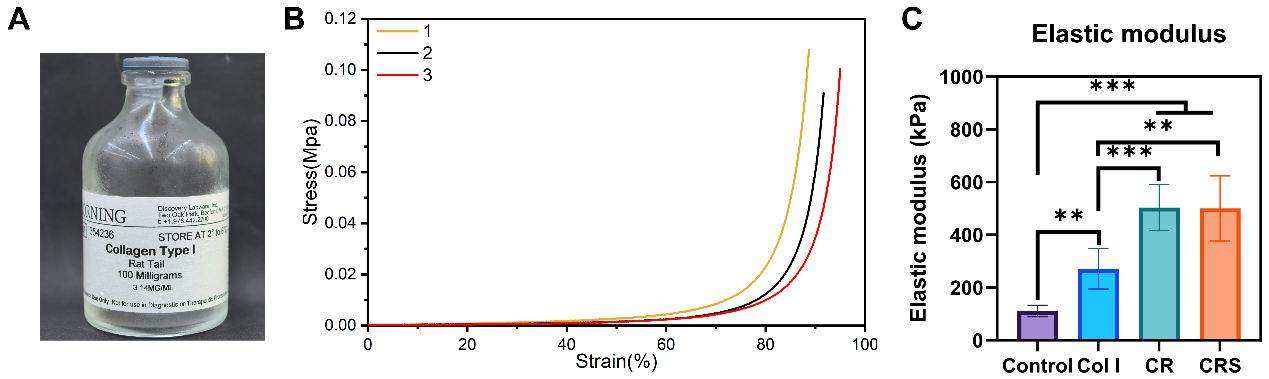
**

**Figure S15.** Elastic modulus of collagen-based scaffolds. A) Commercial rat tail collagen solution, B) Stress-strain curve of rat tail collagen (Corning®), C) Quantified elastic modulus of Col I (Corning®), Col I, CR, and CRS groups.

**Table S1. Primer sequences of RT-qPCR related genes**

| Gene | Peimer(5’-3’) | Length(bp) |
| --- | --- | --- |
| *iNos-F*  *iNos-R*  *Egf-F* | GTTCTCAGCCCAACAATACAAGA  GTGGACGGGTCGATGTCAC  AGAGCATCTCTCGGATTGACC | 127  155 |
| *Egf-R* | CCCGTTAAGGAAAACTCTTAGCA |  |
| *Kgf-F* | CTCTACAGGTCATGCTTCCACC | 174 |
| *Kgf-R* | ACAGAACAGTCTTCTCACCCT |  |
| *CD86-F* | CTGGACTCTACGACTTCACAATG | 131 |
| *CD86-R* | AGTTGGCGATCACTGACAGTT |  |
| *Il1β-F* | TGCCACCTTTTGACAGTGATG | 138 |
| *Il1β-R* | TGATGTGCTGCTGCGAGATT |  |
| *Tnfα-F* | CCCTCACACTCACAAACCAC | 157 |
| *Tnfα-R* | CCCTTGAAGAGAACCTGGGAG |  |
| *Tnfβ-F* | CACTGATACGCCTGAGTGGC | 159 |
| *Tnfβ-R* | TTGGGGCTGATCCCGTTGAT |  |
| *Il10-F* | TGGGTTGCCAAGCCTTATCG | 158 |
| *Il10-R* | GAGAAATCGATGACAGCGCC |  |
| *Arg1-F* | GTACATTGGCTTGCGAGACG | 156 |
| *Arg1-R* | ATCGGCCTTTTCTTCCTTCCC |  |
| *Vim-F* | GCCCTTAAAGGCACTAACGA | 154 |
| *Vim-R* | ATTCACGAAGGTGACGAGCC |  |
| *Fgf10-F* | GTCAGCGGGACCAAGAATGA | 155 |
| *Fgf10-R* | TCGTTGTTAAACTCTTTTGAGCCA |  |
| *Vegf-F* | CGGGCCTCGGTTCCA | 200 |
| *Vegf-R* | GCAGCCTGGGACCACTTG |  |
| *Gapdh-F* | TGGCCTTCCGTGTTCCTAC | 178 |
| *Gapdh-R* | GAGTTGCTGTTGAAGTCGCA |  |
